# Identification of adducts formed between phosphatidylcholine and mustard agents

**DOI:** 10.1007/s00216-026-06600-4

**Published:** 2026-06-13

**Authors:** Matti A. Kjellberg, Noora-Kaisa Rantanen, Shishir Jaikishan, Susanne K. Wiedmer, Hanna Hakulinen

**Affiliations:** 1 Finnish Institute for Verification of the Chemical Weapons Convention (VERIFIN), Department of Chemistry, University of Helsinki, A.I. Virtasen Aukio 1, POB 55, 00014 Helsinki, Finland; 2 Department of Chemistry, University of Helsinki, A.I. Virtasen Aukio 1, POB 55, 00014 Helsinki, Finland

**Keywords:** Biomarker, Chemical warfare agent, Liquid chromatography-mass spectrometry, Mustard gas, Phosphatidylcholine, Phospholipid

## Abstract

Sulphur and nitrogen mustards are two groups of highly toxic blistering agents. Upon human exposure, these agents produce characteristic biomarkers, including alkylated proteins and DNA. In contrast, lipids, which comprise a varied class of biomolecules, have received little attention as potential alkylation targets of mustard agents. In this work, we demonstrate that sulphur and nitrogen mustards bind covalently to 1-palmitoyl-2-oleoyl-glycero-*sn*-3-phosphocholine in model membrane systems. We use ultra-high-performance liquid chromatography-high-resolution tandem mass spectrometry to characterise the structures of these novel analytes. Additionally, alkylated phosphatidylcholines were detectable in human plasma spiked with trace concentrations of sulphur mustard. Plasma samples were prepared by liquid–liquid extraction and analysed by targeted multi-reaction monitoring ultra-high-performance liquid chromatography-tandem mass spectrometry. Our data suggest that these novel alkylated lipids may function as biomarkers of mustard agent exposure.

**Graphical abstract:**

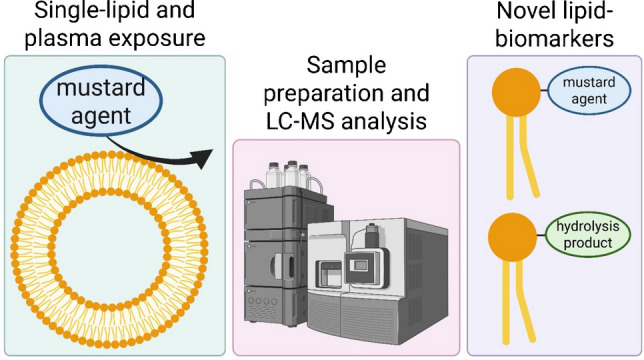

**Supplementary Information:**

The online version contains supplementary material available at 10.1007/s00216-026-06600-4.

## Introduction

**Fig. 1:**
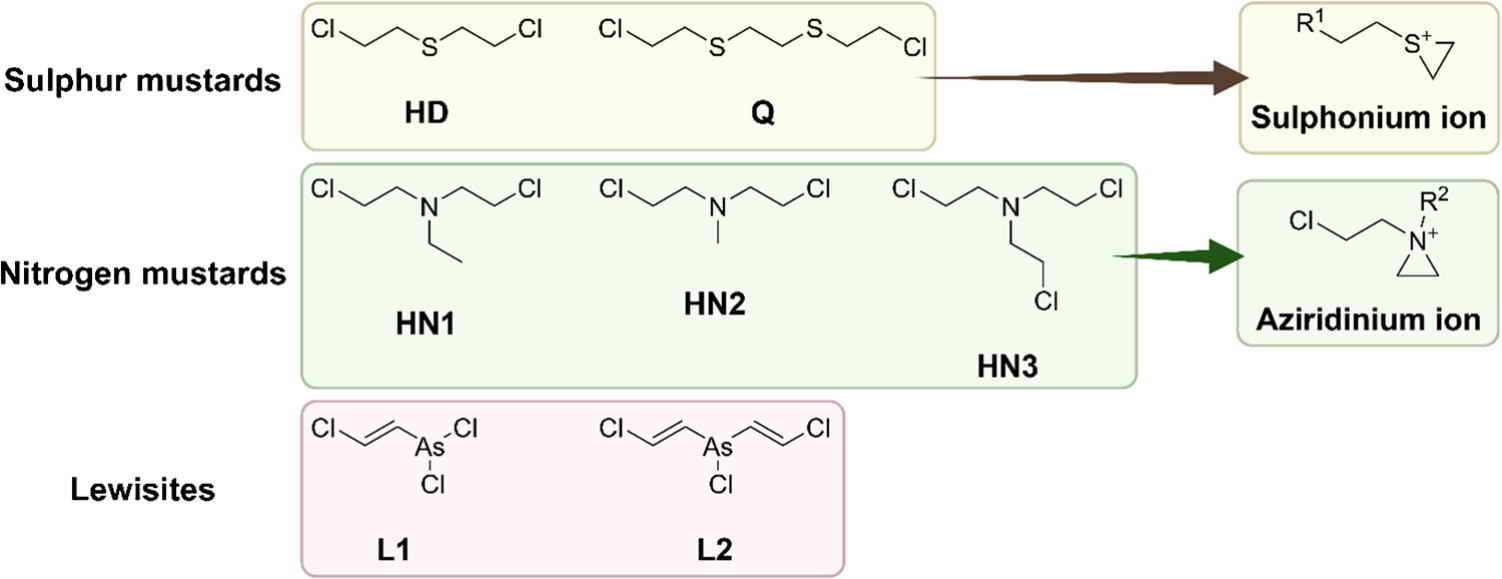
Structures of blistering agents, sulphonium ion, and aziridinium ion. R^1^ is chlorine for HD and -SCH_2_CH_2_Cl for Q. R^2^ is -CH_2_CH_3_, -CH_3_, or -CH_2_CH_2_Cl for HN1, HN2, and HN3, respectively

Mustard agents are highly toxic vesicants with a history of use as chemical weapons [1]. Several mustard agents are classed as Schedule 1 in the Chemical Weapons Convention, and their use is regulated by the Organisation for the Prohibition of Chemical Weapons [2]. Depending on their chemical structures, they are generally grouped as either sulphur or nitrogen mustards (Fig. 1). Their mechanism of cellular toxicity, while widely studied, is not fully understood. In an aqueous environment, the sulphur and nitrogen mustards readily eliminate chloride by intramolecular nucleophilic attack to form cyclic sulfonium and aziridinium ions, respectively (Fig. 1). These ions will potentially alkylate any sterically available nucleophile. In a cellular setting, a variety of biomolecules are alkylated, including glutathione, RNA, DNA and proteins, potentially leading to glutathione depletion, oxidative stress, DNA damage and ultimately programmed cell death [3, 4]. In the long term, mustard agent-induced DNA damage can lead to cancer. Acute symptoms of mustard agent exposure manifest differently depending on dose, exposure mechanism and exposure time [5, 6]. Typical symptoms include skin redness, blistering and necrosis of the skin, irritation of eyes, blindness, shortness of breath and death at higher doses. Symptom onset can be delayed by up to 24 h following exposure, and wound healing is slow relative to ordinary burns. Currently, no antidotes for mustard agents exist.

Lewisites are another class of powerful vesicant chemical warfare agents (CWAs), which, in contrast to mustard agents, contain arsenic (Fig. 1). Lewisites do not have alkylating ability; instead, the trivalent arsenic in the molecule reacts with sulphhydryl groups on various proteins and enzymes. Lewisites function as suicide inhibitors of pyruvate dehydrogenase, a central enzyme in carbohydrate metabolism and cellular respiration [7, 8]. Historically, Lewisites were added to sulphur mustard (HD) to improve the toxicity and lower the freezing point of the resulting CWA mixture [9, 10].

**Fig. 2:**
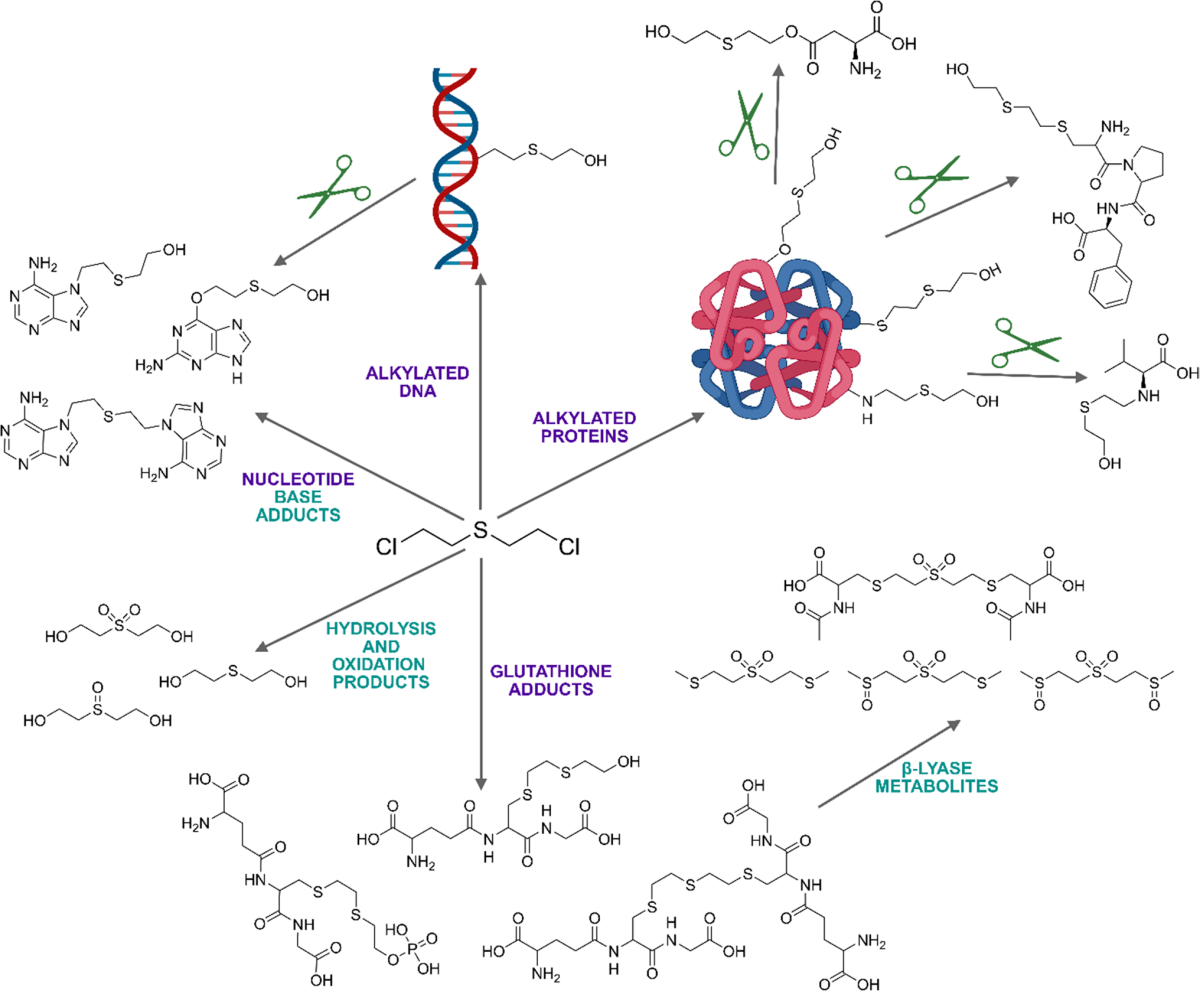
Summary of some of the biomarkers produced by HD. The scissors represent the enzymatic or chemical digestion of macromolecules, which is performed during sample preparation prior to analysis by LC–MS/MS. Biomarkers in purple are found in blood, and biomarkers in turquoise are typically present in urine

Even though the Chemical Weapons Convention restricts the use of mustard agents and Lewisites, exposures in accidents and intentional illicit activities can still happen. The ability to detect and verify the presence of CWAs and their metabolites is essential for exposure verification analysis and is a central focus for the designated laboratory network functioning under the Organisation for the Prohibition of Chemical Weapons. A variety of biomarkers of mustard agent exposure, and the corresponding analysis methods, have been established ([11–17] among others), where the classic mustard agent (HD) has received most of the attention. These biomarkers include mustard agent hydrolysis products, beta-lyase metabolites and excised nucleotide adducts, and are typically quickly excreted in urine [18, 19]. Additionally, mustard agent-specific nucleotide and peptide adducts can be analysed from blood, plasma and hair following extraction and enzymatic or chemical digestion protocols [16, 20–24]. Analysis techniques primarily include targeted gas chromatography‒tandem mass spectrometry (GC–MS/MS) and (ultra-high-performance) liquid chromatography‒tandem mass spectrometry (LC–MS/MS) methods. For GC–MS/MS, the biomarkers generally require additional chemical derivatisation for improved volatility [25–27]. The various biomarkers of HD are summarised in Fig. 2. In contrast to the mustard agents, the biomarkers of Lewisite exposure are scarce. In fact, they are limited only to the hydrolysis and oxidation products of Lewisite 1 and 2 (L1 and L2) [8, 28, 29].

In terms of potential CWA biomarkers, lipids have received relatively little attention. Lipids constitute a major class of biomolecules in all organisms. Together with proteins and carbohydrates, lipids form the backbone of biological membranes that encapsulate all cells, as well as the organelles contained within cells. Additionally, lipids play key roles in many cellular processes, including energy storage, cell signalling and cellular intra- and extracellular transport [30–34]. Lipids have been shown to be targets for chlorine gas, and several chlorinated lipids have been suggested as possible biomarkers of chlorine gas exposure [35–38].

In the literature, lipids are frequently mentioned as potential targets of mustard agents, but experimental evidence demonstrating the direct alkylation of lipid molecules is sparse. Lüling et al. [39] showed that HD alkylates steroid hormones in ethanolic solutions. However, to the best of our knowledge, alkylation of major cellular and plasma lipids (such as glycerophospholipids and cholesterol) in biologically relevant matrices has not been reported.

Here, we demonstrate that several sulphur and nitrogen mustards bind covalently to 1-palmitoyl-2-oleoyl-glycero-*sn*-3-phosphocholine (POPC), using an aqueous model lipid membrane system. The reactivity of Lewisites with POPC is also investigated. POPC was chosen as the target lipid because it is commercially available, relatively inexpensive and ubiquitously present in natural lipid membranes [34, 40, 41]. In an aqueous environment, the amphiphilic nature and cylindrical shape of POPC orient the lipid molecules into bilayer formation, mimicking the structure of natural cell membranes.

We also show that alkylated phosphatidylcholines (PCs) are detectable in human plasma that has been spiked with trace amounts of HD. These findings pave the way for a potential new type of mustard agent biomarker. The possible contribution of these modified phospholipids to the overall pathology of mustard agent exposure is an interesting topic for future research.

## Materials and methods

### Reagents and chemicals

2-Propanol (IPA; high-performance liquid chromatography (HPLC) grade), methanol (MeOH; LC–MS grade), dichloromethane (DCM; HPLC grade) and acetone (HPLC grade) were purchased from Fisher Chemicals (Hampton, NH, USA). LC–MS-grade acetonitrile (ACN) was bought from Honeywell (Charlotte, NC, USA). A Millipore Direct-Q®3 UV system (Merck; Darmstadt, Germany) was used to produce ultra-pure water. Ammonium formate (AmF) (1 M (63.0 g/l) solution) and ammonium acetate (AmAc) (1 M (77.1 g/l) solution) used in the LC mobile phases were prepared by dissolving AmF (99.995%; Aldrich; Darmstadt, Germany) and AmAc (99.995%; Honeywell) salts in ultra-pure water. LC–MS-grade formic acid (FA; 99%) was purchased from Sigma-Aldrich (Darmstadt, Germany). Human plasma (FFP24, produced from citrate-anticoagulated blood, ISBT A0081V00/E3947) was acquired from the Finnish Red Cross.

A standard solution of POPC in chloroform (25 mg/ml (32.9 mM)) was obtained from Avanti® Polar Lipids (Alabaster, AL, USA). For the experiments, a 10 mM (1.6 mg/ml) solution of POPC was prepared by evaporating adequate amounts of the lipid stock solution to dryness and reconstituting it in ultra-pure water.

HD (bis(2-chloroethyl)sulphide; 95%) and L1 (2-chlorovinylarsinedichloride; > 90%) were received from the Finnish Defence Research Agency (Lakiala, Finland). Nitrogen mustard 1 (HN1; bis(2-chloroethyl)ethylamine; > 90%), nitrogen mustard 2 (HN2; bis(2-chloroethyl)methylamine; > 90%), nitrogen mustard 3 (HN3; tris(2-chloroethyl)amine; > 90%), and Lewisite 2 (L2; bis(2-chlorovinyl)arsine chloride; 97%) were obtained from Spiez Laboratory (Spiez, Switzerland). Sesquimustard (Q; 1,2-bis((2-chloroethyl)thio)ethane; 90%) was synthesised at the Finnish Institute for Verification of the Chemical Weapons Convention (VERIFIN). Thiodiglycol (TDG; 90%; VERIFIN, Finland), bis(ethanol)ethylamine (EDEA; 94%; Aldrich), bis(ethanol)methylamine (MDEA; 99%; Aldrich), and tris(ethanol)amine (TEA; 99%; Aldrich) were synthesised at VERIFIN or bought from commercial suppliers. Stock solutions of the blistering agents (HD, Q, HN1, HN2, HN3, L1, and L2) and degradation products (TDG, EDEA, MDEA, and TEA) were prepared by dissolving adequate amounts of pure chemical in acetone. A stock solution of 1,2-bis(2-hydroxyethylthio)ethane (Q-diol) was prepared by mixing one volume of Q stock solution with one volume of ultra-pure water and allowing the mixture to stand for several days. Stock solutions of the degradation products of L1 and L2 (2-chlorovinylarsonic acid (CVAOA) and bis(2-chlorovinyl)arsinic acid (b-CVAOA), respectively) were prepared by spiking five volumes of L1 and L2 stock solution with one volume of 33% hydrogen peroxide (H_2_O_2_; VWR International; Radnor, PA, USA). The structures of the blistering agents and their degradation products, and the concentrations of the stock solutions used in the experiments are listed in Table 1. The presence of the target blistering agents and their degradation products was confirmed with GC–MS/dual flame photometric detection or LC–MS/MS prior to spiking. Reference chemicals of the alkylated POPCs were synthesised according to methods described in “Single-lipid exposure experiments”. Because the concentrations of the target analytes were sufficient for the experiments, the crude products were used without additional purification.

**Table 1:**
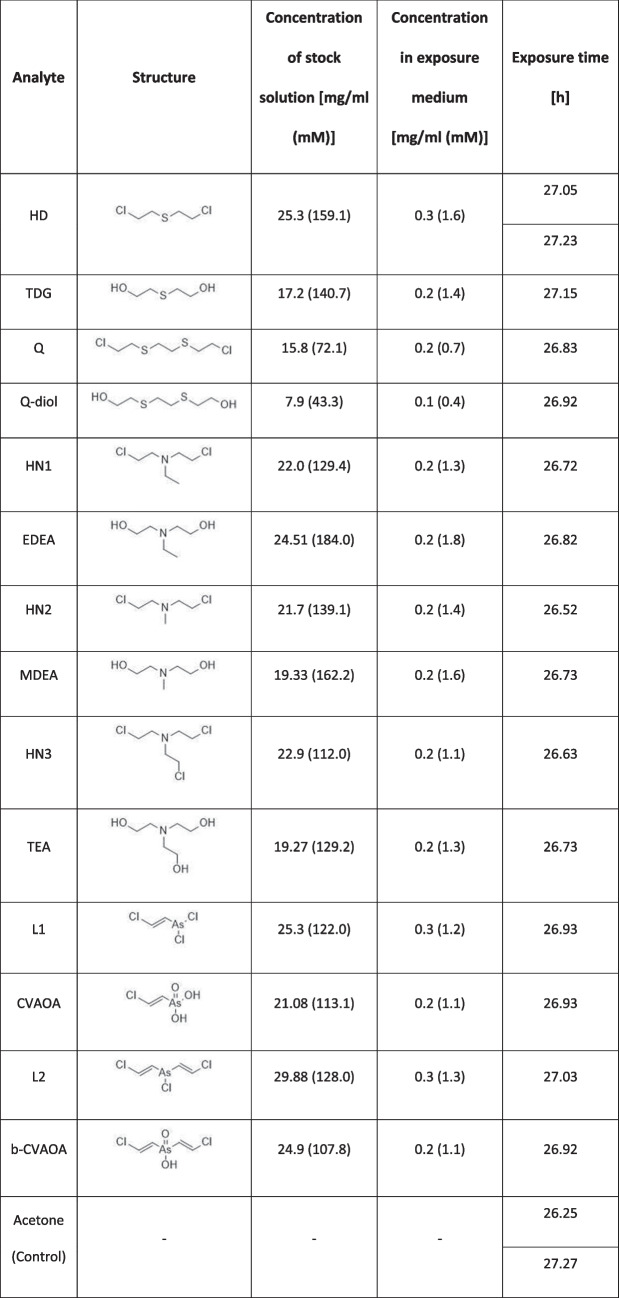
Blistering agents and their degradation products used in this study and the lipid exposure samples prepared from them

### Instruments and methods

#### Liquid chromatography high-resolution mass spectrometric analyses

Untargeted ultra-high-performance liquid chromatography‒high-resolution mass spectrometry (LC-HRMS) screening of the lipid samples was performed using a Thermo Fisher Scientific Dionex Ultimate 3000 UHPLC system coupled to a Thermo Fischer Scientific Orbitrap Fusion™ MS (Waltham, MA, USA). The MS was equipped with a heated electrospray ionisation (HESI) source. The ion transfer tube temperature was set at 230 °C and vaporiser temperature at 350°C. Sheat gas, aux gas and sweep gas flows were 40, 15 and 0 (arbitrary units), respectively. The spray voltage was 4000 V. Scanning was done over mass-to-charge ratio (*m*/*z*) 600‒1000 in positive polarity with the resolution setting 60 000 at *m*/*z* 200. Ions detected in the screening were manually evaluated based on their accurate masses and isotopic patterns, after which LC–MS/HRMS with high-energy collisional dissociation (HCD) was performed to obtain structural information on interesting ions. The stepped fragmentation function was used to yield fragmentation patterns of relative HCD collisional energies of 15%, 30%, and 45% in a single run. LC–MS/MS/HRMS (MS^3^) experiments were performed with HD-POPC, to verify the structure of the identified adduct. The ion corresponding to HD-POPC (*m*/*z* 882.58078) was isolated in MS^1^ and collided to produce the fragment ions at *m*/*z* 306.06902 (HCD 35%), 278.03772 (HCD 40%), and 242.06104 (HCD 40%), all of which were then further collided in MS^2^ (HCD 35%) to produce fragmentation patterns of the isolated ions (full scan with ranges *m*/*z* 48–316.06, *m*/*z* 46–288.03, and *m*/*z* 44–252.06, respectively). The mass resolution in the LC–MS/MS/HRMS experiments was set at 60,000 and at 120,000 (at *m*/*z* 200) for the LC–MS/HRMS experiments.

Reverse-phase LC separation using a Waters™ XBridge® BEH C18 (length 100 mm, inner diameter 2.1 mm, particle size 2.5 µm) column was used for the HRMS-based analyses. The column temperature was set at 60°C. The mobile phases consisted of 5 mM (0.3 g/l) AmF and 0.1% FA in 60% ACN/ultra-pure water (eluent A), and 5 mM (0.3 g/l) AmF and 0.1% FA in 80% IPA/20% ACN (eluent B) (all percentages by volume). Flow rate was set to 0.6 ml/min, and the injection volume was 5 µl. A 20-min gradient elution method was used: hold 5% B for 0.6 min, linear increase to 100% B in 12.4 min, hold 100% B for 4 min, linear decrease to 5% B in 1 min, and hold 5% B for 2 min. A slightly modified chromatographic method, where the IPA in eluent B was replaced with MeOH, was used in the analysis of the fragmentation patterns of the molecular ion ([M+H]^+^) and the two first isotopic peaks ([M+1+H]^+^ and [M+2+H]^+^) of HD-POPC from a synthesised reference sample (“Single-lipid exposure experiments”).

Plasma samples exposed to 100 ng/ml (0.6 µM), 10 µg/ml (0.1 mM), and 250 µg/ml (1.6 mM) of HD (“HD exposure of plasma and subsequent lipid extraction”) were analysed using the reverse-phase (MeOH eluents) and hydrophilic interaction liquid chromatography (HILIC). The analytical column used in the HILIC analysis was a Waters™ AQUITY UPLC® BEH HILIC (length 100 mm, inner diameter 2.1 mm, particle size 1.7 µm) operated at 45 °C. Eluent A consisted of 10 mM (0.8 g/l) AmAc in 95% ACN/5% ultra-pure water (percentages by volume), and eluent B of 10 mM (0.8 g/l) AmAc in 50% ACN/ultra-pure water (by volume). Flow was set at 0.5 ml/min and the gradient had the 16 min elution program had the following steps: 100% A for 1 min, linear decrease to 80% A in 11.5 min, hold at 80% A for 1 min, linear increase to 100% A in 0.5 min, and hold at 100% A for 2 min.

#### Liquid chromatography unit-resolution tandem mass spectrometric analyses

A Waters™ Xevo® TQ-XS triple quadrupole mass spectrometer equipped with a Waters™ Aquity I-Class UPLC® chromatographic separation system (Milford, MA, USA) was used in the analysis of the extruded vesicle samples and the exposed plasma samples (see “POPC vesicle extrusion and HD exposure” and “HD exposure of plasma and subsequent lipid extraction”). Ions were produced using electrospray ionisation (ESI) in positive mode. The following instrument settings were used: source temperature 150 °C, desolvation temperature 500 °C, cone voltage 20 V, and desolvation gas (N_2_) flow 1000 l/h. Capillary voltage was set at 3500 V (positive mode). Precursor ion scanning of *m*/*z* 184 in the scan range of *m*/*z* 600–1000, with collision energy of 33 V, was used to collect PC-lipidome data of plasma samples. Multi-reaction monitoring (MRM) methods were developed for select target analytes. Analysis parameters for the MRM methods are presented in Supplementary Information (SI) B Table B.1.

A different HILIC method was used in the LC‒MS/MS analysis of plasma samples. The column temperature was 45 °C and mobile phases were ACN (eluent A) and 55 mM (3.5 g/l) AmF in 60% ACN/40% ultra-pure water (eluent B) (all percentages by volume). For the first minute, the mobile phase contained 2% of eluent B. Within the next 9 min, the amount of eluent B was linearly increased to 20%, followed by a linear increase to 40% of eluent B within 2 min. The amount of eluent B was held at 40% for 3 min, after which it was linearly decreased back to 2% in 1 min and allowed to equilibrate for 4 min. The total run time of the gradient elution method was 20 min. The flow was 0.5 ml/min and the injection volume was 5 µl.

### Single-lipid exposure experiments

Fifteen POPC vesicle samples were prepared for the single-lipid exposure experiments. Samples with 500 µl volumes and concentrations of 1 mM (0.2 mg/ml), were diluted from a 10 mM (1.6 mg/ml) POPC water stock solution in 10 ml KIMAX™ (Merck) screw-capped glass test tubes, and the suspensions were sonicated in a bath-sonicator (Branson Ultrasonics™ Bransonic™ ultrasonic cleaner B200; Branson Ultrasonics, Brookfield, CT, USA) for 20 min at room temperature. Immediately after sonication, 5 µl of blistering agent or degradation product stock solution was added to the vesicles. 5 µl of acetone was added to the control samples. The samples were briefly vortexed after addition of the analytes or vehicle solvent, and then left at room temperature to react overnight. The analyte concentrations in the exposed samples and the exposure times are presented in Table 1. The mean exposure time was 26.84 ± 0.25 h. A modified Folch lipid extraction procedure (liquid–liquid extraction of samples using water, MeOH and chloroform) was applied [42] to extract the samples: 0.5 ml of ultra-pure water, 1.5 ml of MeOH and 3 ml of DCM were added to the test tube. The samples were vortexed vigorously for 15‒30 s and then agitated manually for 2 min. The solvent phases were allowed to separate by gravity, after which approximately 90% of the DCM layer was collected. The DCM layers were evaporated to dryness under mild nitrogen flow at 35 °C (Caliper TurboVap® LV Evaporator; Hopkinton, MA, USA), and the residues were dissolved in 1 ml of IPA. The samples were diluted by a factor of ten using IPA (corresponding to approximately 50 µM of POPC starting material) before analysis by LC-HRMS and LC–MS/HRMS. Each exposure experiment was repeated at least once, with similar results. For the analysis of the fragmentation patterns of the isotopic peaks of HD-POPC, a 0.5 mM (0.08 mg/ml) POPC sample was spiked with 5 µl of HD stock solution and extracted as described above.

### POPC vesicle extrusion and HD exposure

Four replicates of 1 mM (0.2 mg/ml) POPC vesicle solutions (1 ml each) were prepared from a 10 mM (1.6 mg/ml) stock solution using ultra-pure water as the diluent. Three of the samples were subjected to extrusion to generate unilamellar vesicles of size 100 nm, 50 nm and 30 nm, respectively. The prepared lipid dispersions were extruded 11 times through Millipore polycarbonate filters (Bedford, MA, USA) with respective pore sizes (100 nm, 50 nm, and 30 nm) using an Avanti MiniExtruder (Avanti Polar Lipids, Alabaster, AL, USA) to yield uniform unilamellar vesicles. The size (hydrodynamic diameter) distribution of the extruded vesicles was validated using dynamic light scattering with a Zetasizer Nano ZS instrument (Malvern Instruments, Malvern, Worcestershire, UK). For comparison, one sample was bath-sonicated as described previously ("Single-lipid exposure experiments") and its average vesicle size was measured. The size extruded and sonicated samples were exposed to HD and extracted, as described previously ("Single-lipid exposure experiments"). The experiments were repeated twice (n = 3). The extracted samples were analysed by LC–MS/MS, utilising MRM. The signal intensities of HD- and TDG-POPC in the samples were measured and normalised to the signal intensity of unreacted POPC, for each sample. Optimised MRM methods were constructed for HD- and TDG-POPC. As the levels of unreacted POPC in each sample exceeded the alkylated analytes by several 100-fold, a suboptimal MRM method was constructed for POPC. This was done to avoid the risk of saturation of the POPC signal and the subsequent skewing of the acquired results. The method parameters are presented in SI B Table B.1.

### HD exposure of plasma and subsequent lipid extraction

Human plasma was exposed to 100 ng/ml (0.6 µM) HD by spiking 5 µl of a 10 µg/ml (63 µM) HD stock solution in acetone into 500 µl of plasma at room temperature. A vehicle control was prepared by spiking 5 µl of pure acetone to 500 µl of plasma. The samples were vortexed thoroughly immediately after spiking and allowed to react at 37 °C overnight with mild agitation (300 rpm). The incubated samples were subjected to extraction by a modified Folch extraction protocol [42]. Two hundred microliters of the HD-exposed plasma sample was transferred to a 10 ml KIMAX™ screw cap glass test tube and diluted to 2 ml with ultra-pure water. Next, 3 ml of MeOH was added, followed by 6 ml of DCM. The sample was vortexed vigorously for 15–30 s, followed by shaking for 2 min. The solvent phases were separated by centrifugation (2000 × g for 5 min at room temperature), and approximately 90% of the lower (DCM) solvent layer was collected. The collected DCM layer was dried under mild nitrogen flow at 35 °C, after which the dry residue was reconstituted in 200 µl of IPA and transferred to an analysis vial, prior to analysis by LC–MS/MS. The plasma exposure experiments were repeated once with similar results.

For the LC-HRMS and LC–MS/HRMS analyses, a high-exposure sample (250 µg/ml (1.6 mM)) was prepared by spiking 5 µl of a 25.3 mg/ml (159.1 mM) to 500 µl. The sample was incubated and extracted as described above, but the evaporation residue of the DCM-extract was reconstituted in 200 µl MeOH for analysis. To test the instrument sensitivity, a more diluted sample (10 µg/ml (63 µM)) was prepared by diluting the high-exposure sample extract with untreated plasma lipid-extract.

## Results and discussion

### Identification of POPC-blistering agent adducts

A single-lipid model membrane system, prepared in ultra-pure water, was initially chosen over exposure to a biological matrix (e.g., blood or plasma). This was due to the inherently more straightforward analysis attributed to the former, as it is estimated that a living cell may contain approximately 1000 distinct lipid molecular species [43–45], in addition to a plethora of other reactive biomolecules.

POPC vesicles were exposed to selected blistering agents as well as their hydrolysed analogues (Table 1). Lewisites L1 and L2 were chosen in addition to mustard agents, as they are vesicant CWAs and have historically been used together with HD in CWA mixtures [9, 10]. The lipid samples were analysed by LC-HRMS and LC–MS/HRMS using a C18 silica RP column after exposure and subsequent extraction. All selected mustard agents generated covalently modified POPCs (Table 2). None of the corresponding hydrolysis products reacted with POPC, nor were we able to identify Lewisite (L1 and L2)-modified lipids. This was not unexpected since neither Lewisites nor hydrolysis products of sulphur and nitrogen mustards have alkylation capability.

**Table 2:**
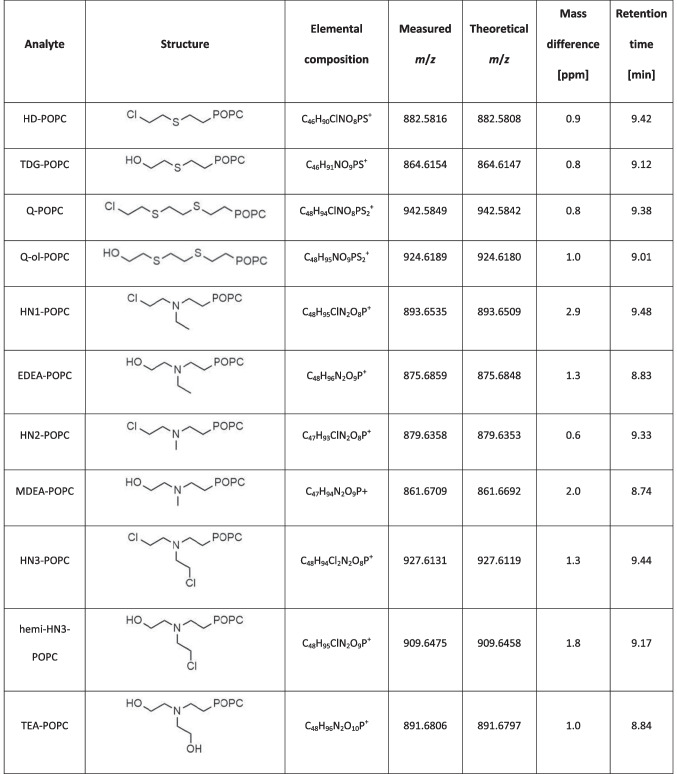
Identified POPC blistering agent adducts. Mass difference is presented as reported by the instrument software

**Fig. 3:**
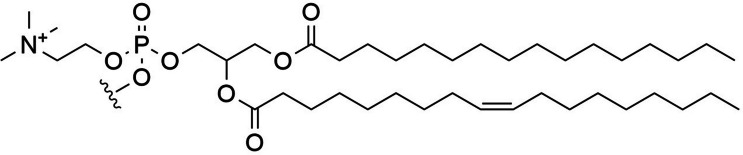
Structure of the POPC substituent. All mustard agents alkylated the hydroxyl group in the phosphocholine headgroup of POPC. In Table 2, the POPC substituent in the adducts is abbreviated as POPC

Two criteria were used in the identification of POPC adducts. Firstly, the measured accurate *m*/*z* values were not to differ more than 5 ppm from the predicted theoretical *m*/*z* of the alkylated POPC for both precursor and fragment ions. Secondly, the fragmentation patterns were to have logical similarities with the fragmentation of the unmodified phospholipid and the blistering agent or the degradation product in question. Eleven analytes were identified using these criteria. The analytes were the POPC adducts of HD, Q, HN1, HN2 and HN3 and their hydrolyzed analogues. The partially hydrolyzed adduct of HN3 (hemi-HN3-POPC) was also identified. Based on the fragmentation patterns, the blistering agents were determined to be bound to one of the oxygen atoms in the phosphocholine headgroup (Fig. 3). According to literature, POPC mainly fragments through loss of the phosphocholine headgroup [46], which was confirmed in our LC–MS/MS analyses of the blank vesicle samples. The POPC adducts fragmented similarly, where the main fragments included the corresponding alkylated phosphocholine. LC–MS/MS/HRMS (MS^3^) experiments were performed on the alkyl-phosphocholine fragment produced from HD-POPC (*m*/*z* 306.06902) as well as its two subsequent fragments (*m*/*z* 278.03772 and *m*/*z* 242.06104) to verify the site of the alkylation. In particular, the *m*/*z* 182.98754 fragment produced by colliding *m*/*z* 242.06104 demonstrates that the only feasible site of alkylation is one of the oxygens in the phosphate (Tables A.2–A.4 in SI A).

The 11 identified adducts are summarised in Table 2, where the POPC substituent in the adducts is abbreviated as POPC. The complete structures and their HRMS fragmentation patterns are presented in Tables A.1–A.14 in SI A.

**Fig. 4:**
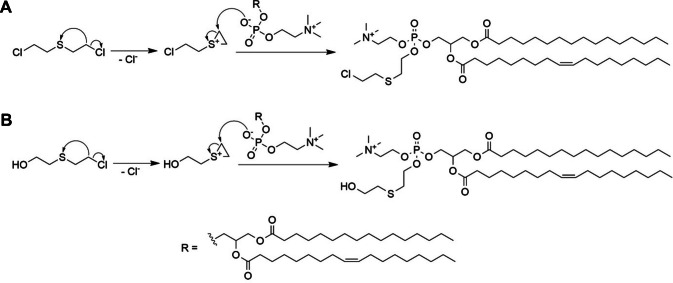
Reaction mechanism of the alkylation of POPC by HD (**A**) and hemisulphur mustard (**B**)

Mustard agents are strong alkylating agents that react with any sterically available nucleophile. Therefore, it is not unexpected that they alkylate the phosphate in the phosphocholine headgroup of POPC, especially since the phosphocholine headgroups of the POPC molecule are oriented towards the aqueous solvent in a bilayer vesicle structure. Additionally, sulphur and nitrogen mustards have previously been shown to alkylate free phosphate in aqueous solution [47, 48]. Unexpectedly, in our experiments, we observed that the most prevalent analytes produced were the POPC adducts retaining the chlorine(s) at the end of the mustard tail. The hydrolysed and partially hydrolysed POPC adducts (e.g., TDG-POPC and hemi-HN3-POPC, Table 2) were also detected, however, to a markedly lesser degree (based on signal intensity). Considering the length of the exposure time (26.84 ± 0.25 h on average), one would expect that complete hydrolysis of the mustard tail in the POPC-adducts would have occurred, as free mustard agents are known to rapidly hydrolyse in aqueous solution [49]. Consequently, the chlorine-containing POPC adducts can be assumed to be quite stable. While no specific stability experiments were conducted for this work, HD-POPC signal intensity did not appreciably degrade as measured by LC–MS/MS in aqueous solution over several days at 4 °C (data not shown). The stability of the chlorine-containing analogues suggests that the hydrolysed and partially hydrolysed adducts are more likely produced through partially hydrolysed mustard agent intermediates (e.g. hemisulphur mustard) that retain alkylating capability, rather than through further hydrolysis of the POPC adducts (Fig. 4).

The reason for the apparent stability of the chlorine-containing POPC adducts is open for speculation. One possibility is that the persistent positive charge on the quaternary ammonium group attracts the electronegative chlorine at the end of the mustard tail, stabilising its structure and inhibiting formation of the reactive sulphonium/aziridinium intermediates. Alternatively, the mustard tail might be protected from hydrolysis by being inserted between the lipid bilayer membrane. No crosslinked POPCs were observed in any of the experiments, suggesting that the mustard tail does not react further after alkylation of the phosphocholine headgroup.

### Effect of POPC vesicle size on rate of alkylation by HD

Next, the study investigated how POPC vesicle size influences the rate of alkylation by HD, utilising an aqueous model membrane system to evaluate the impact of lipid bilayer curvature. Bath sonication and extrusion methods were used to generate vesicles of different sizes and consistency. Bath sonication generally produces polydisperse vesicle populations [50], whereas shorter sonication times (< 10 min) result in larger, multilamellar structures [51] and longer sonication times (20–60 min) yield smaller diameters [52]. Woodbury et al. characterised the emergence of a bimodal distribution of vesicles, for both 30 s of mild sonication as well as for 1 h of long duration [52]. This study utilised a “standard” 20-min bath sonication (as described in “Single-lipid exposure experiments” and “POPC vesicle extrusion and HD exposure”) to produce non-uniform vesicles with broader size distribution, including some multilamellar vesicles [53]. It should be noted that 20 to 60 min of bath sonication is still the conventional method to create vesicles of various sizes and lamellarity. These discrepancies in the processing time are likely attributable to variations in total lipid mass and sample concentration (and volume), which significantly influence the efficiency of ultrasonic energy transfer.

**Fig. 5:**
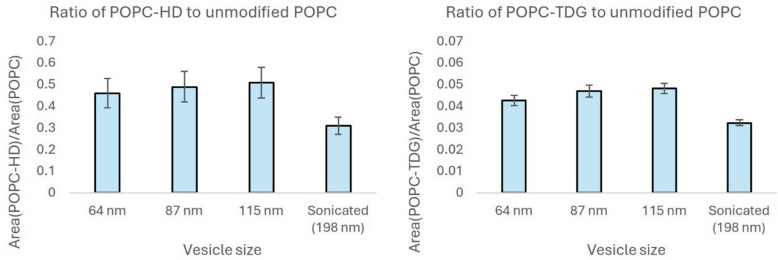
Production rate of HD- and TDG-POPC in differently sized extruded uniform vesicles (64, 87, 115 nm), compared with a sonicated sample (average vesicle size 198 nm). Left: HD-POPC, right: TDG-POPC

To isolate the specific effects of membrane curvature on alkylation, size extrusion was used to produce uniform POPC vesicles of distinct diameters for HD exposure testing. Three extruded POPC vesicle dispersions of equal concentration (1 mM (0.2 mg/ml), extruded through 30, 50 and 100 nm filter), as well as a bath-sonicated sample (1 mM (0.2 mg/ml)), were exposed to equimolar amounts of HD. The actual sizes of the vesicles in the extruded samples were measured by dynamic light scattering and were determined to be 64 nm, 87 nm and 115 nm, respectively. In lipid systems, it is a common observation that the final vesicle diameter does not perfectly match the nominal pore size of the polycarbonate membrane. This discrepancy is driven by the concentration of the sample, number of extrusion cycles, physics of bilayer deformation, pressure applied during the process and elastic properties of the lipids used. The resulting vesicles are often slightly larger (e.g. ~ 110–130 nm for 100 nm pore). This occurs because the lipid bilayer has a finite bending rigidity. The sonicated sample had an average vesicle size of 198 nm. After exposure, samples were extracted and analysed by LC–MS/MS, utilising MRM. The signals of HD-POPC and TDG-POPC were normalised to the signal of unreacted POPC for each sample and compared with each other (Fig. 5).

The data show that the vesicle size does not have any significant effect on the degree of alkylation under the tested conditions. However, the extruded samples demonstrated a significantly higher degree of alkylation than the sonicated sample. It is possible that some of the lipid membrane structures produced by sonication are not as susceptible to alkylation, and the sonicated sample may also contain multilamellar vesicles that affect the alkylation rate. Larger and multilamellar vesicles logically present an overall reduced surface area relative to smaller vesicles with which HD can interact. Additionally, membrane curvature may play a role in the alkylation rate, where too large or too small vesicle structures may present suboptimal steric availability. More in-depth analyses should be conducted to elucidate the physicochemical properties of vesicle composition, size and uniformity affecting the mustard agent alkylation rates.

### Suitability of HD-alkylated phosphatidylcholines as biomarkers of exposure in human plasma

While a single-lipid model system is a good starting point for studying the possible formation of mustard agent-alkylated lipids, exposure of biologically relevant matrices is necessary for testing the viability of these lipids as biomarkers. Biological tissues, including blood and plasma, contain a myriad of lipid species, comprising many different PC molecular species with varying acyl chain compositions and distinct molecular masses. Thus, selecting a probable PC molecular species that is alkylated requires knowledge of the overall PC composition of the exposed tissue. While POPC is present in most tissue, it is not necessarily the most prominent PC [54] and may therefore not be the optimal lipid to consider as a target for alkylation.

**Fig. 6:**
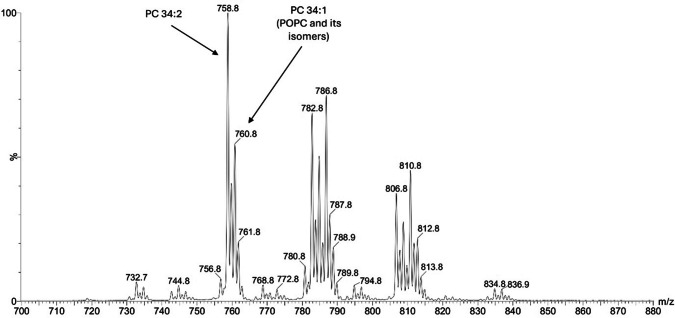
Par184 combined mass spectrum of HD-exposed plasma. The spectrum demonstrates the PC lipidome of the plasma batch used in this work. PC 34:2 and PC 34:1 are denoted. The spectrum is combined from RT 11.50–12.60 min from chromatogram in SI B, Fig. B.1

Here, as a proof-of-concept experiment, human plasma was exposed to the classic mustard agent HD at trace levels (100 ng/ml, 0.6 µM). To elucidate the most likely HD-alkylated PC (HD-PC) produced, a positive precursor (parent) ion scan of *m*/*z* 184 (denoted here as Par184) of the exposed plasma sample was performed. Par184 is an MS^2^-scanning method that is specific for phosphocholine-containing lipids (e.g. PC and sphingomyelin) and can be utilised in lipidomic MS-analysis [55, 56]. Based on the Par184 data, the most prominent PC in the plasma sample was PC 34:2 (*m*/*z* 758.8, Fig. 6). POPC and likely its isomers (PC 34:1, *m*/*z* 760.8) were also present in significant amounts. To achieve maximum sensitivity of detection, MRM methods were constructed for the potentially HD-alkylated versions of these PCs (here denoted HD-PC 34:1 and HD-PC 34:2). The MRM transitions were chosen based on common HD-PC fragments (SI A, Table A.1) using the predicted theoretical *m*/*z* of the corresponding HD-PC as the precursor (*m*/*z* 882.6 and 880.6 for HD-PC 34:1 and HD-PC 34:2, respectively). Collision energies and other MRM parameters were previously optimised based on HD-POPC (*m*/*z* 882.6) that was produced in the extruded lipid vesicle experiments (see “POPC vesicle extrusion and HD exposure”). As variations in acyl chain composition of different PC molecular species should not markedly affect the headgroup-derived fragmentation characteristics, the same collision energies were chosen for both HD-PCs (SI B, Table B.1).

**Fig. 7:**
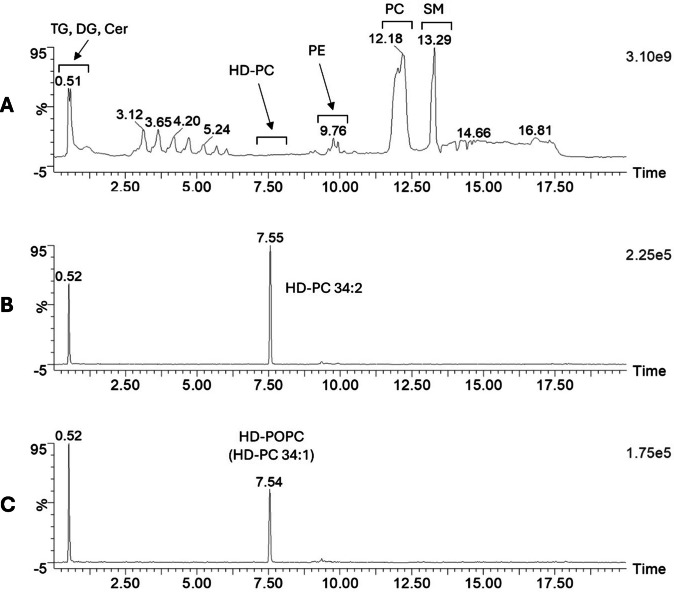
HILIC chromatograms of the trace-level HD-spiked plasma sample. **A** MS positive full scan analysis (*m*/*z* 600–1000) showing the different retention times of common plasma lipids ionisable in positive mode under the conditions used. **B** MRM TIC of HD-PC 34:2. **C** MRM TIC of HD-PC 34:1. TG, triglycerides; DG, diglycerides; Cer, ceramide; PE, phosphatidylethanolamine; PC, phosphatidylcholine; SM, sphingomyelin; HD-PC, HD-alkylated phosphatidylcholine. Similar LC-HRMS chromatograms are presented in Fig. B.4. in SI B

Initially, RPLC (silica C18) was attempted in the analysis of the plasma samples, but no HD-PCs could be detected in these experiments. With RPLC, the amphiphilic PCs are separated mainly based on the composition of their non-polar acyl chains. As the HD modification occurs in the phosphocholine headgroup, the retention times of the HD-PCs are not necessarily markedly changed from that of their unmodified counterparts, and the retention times may therefore overlap. As the overwhelming majority of PCs in the exposed sample remain unmodified, HD-PCs are likely to suffer from signal suppression in the ion source due to co-elution. To ensure that HD-PCs do not go undetected due to signal suppression, HILIC was used in the analysis. HILIC works in the opposite manner to RPLC. Here, the lipids are retained on the column sorbent based on their polarity, where less polar headgroup-containing lipids are eluted earlier [57]. Alkylation of the phosphocholine headgroup reduces the overall headgroup polarity, which means that HD-PCs will elute earlier than their unmodified counterparts. Consequently, the modified lipids are less likely to suffer from signal suppression. Using HILIC separation and MRM-detection, both HD-PCs could be detected in the HD-exposed sample (Fig. 7, SI B, Fig. B.2), and, based on MS full-scan analysis of plasma samples, the HD-PCs eluted at a retention time with no detectable, co-eluting interfering analytes (Fig. 7). This is in good agreement with knowledge on the retention mechanisms in HILIC and demonstrates that HILIC separation is particularly useful for HD-PC detection, when studying complex lipid samples [57]. Par184 and HD-PC specific Par306 scanning was unsuccessful in detecting HD-PCs using HILIC, likely due to low levels of target lipids in the sample. The HILIC-MRM method applied here did not resolve the two HD-PCs chromatographically. Consequently, some of the HD-PC 34:1 signal observed in the MRM analysis is likely derived from the [M+2+H]^+^ isotope of HD-PC 34:2, which shares the same unit m/z value (i.e. 882.6). LC–MS/HRMS fragmentation experiments using HD-exposed POPC were conducted to investigate the contribution of the [M+2+H]^+^ isotope to the measured HD-PC 34:1 signal. Fragmenting the [M+2+H]^+^ isotope yielded the same product ions as the monoisotope mass, however, to a markedly lesser degree (SI B, Fig. B3). Many of the product ions will contain the ^37^Cl or ^34^S isotopes, have a *m/z* shift of +2, and will therefore not interfere with the MRM signal. Additionally, the presence of two different alkylated PCs (HD-PC 34:1 and HD-PC 34:2) was verified from plasma samples spiked with higher levels of HD (10 and 250 µg/ml (63 µM and 1.6 mM)) using LC–MS/HRMS (SI B, Fig. B.4 and B.5). No HD-PCs were detected in the untreated control sample in the unit- (SI B, Fig. B.2) and high-resolution LC–MS/MS analyses.

The limits of detection for the selected HD-PCs extracted from spiked plasma samples were not established as part of this work. However, the fact that HD-PCs could be detected with relative ease after low-level (100 ng/ml (0.6 µM)) plasma exposure, without extensive optimisation of sample preparation or analytical methods, is promising. This suggests that mustard agent-alkylated PCs could be suitable plasma-derived biomarkers, perhaps comparable to serum albumin adducts, which can be detected in plasma and serum samples exposed to low ppb levels of mustard agents [16]. In future studies, more thorough sample preparation and analysis method development should be conducted and validated to optimise the limits of detection of mustard agent-alkylated PCs.

Analyte detection limit is not the only parameter for determining a good biomarker [58]. Some excellent mustard agent biomarkers (such as haemoglobin and serum albumin adducts) can persist in the body for several weeks to months post-exposure [59, 60]. In general, the cellular turnover of phospholipids is rather rapid. For example, the half-life of PC in proliferating cells is mere hours [61, 62]. Whether or not alkylation of the PC headgroup affects its turnover is a question that remains to be answered, and the in vivo stability and possible metabolic conversion of alkylated PCs should be investigated. Additionally, it is not far-fetched to speculate that alkylation of a phospholipid headgroup could affect lipid-mediated cellular processes such as the generation of lipid-derived signalling molecules (e.g. phosphocholine, phosphatidic acid, diacylglycerol and arachidonic acid) [31, 63, 64]. Indeed, the possible role that lipid-alkylation plays in the overall pathophysiology of mustard agents opens interesting new avenues of research.

## Conclusions

Comparatively little research has focused on lipids as potential targets for CWAs. Chlorine gas has been shown to modify a variety of lipids both in vitro and in vivo [36, 37, 65–68]. Although, in the literature, lipids are frequently identified as potential targets for mustard agent alkylation, there is a notable absence of publications providing *de facto* evidence of such modifications within common biomembrane lipids.

In this work, we used LC-HRMS-based methods to demonstrate that various mustard agents alkylate POPC in aqueous model membrane systems. All selected mustard agents—HD, Q, HN1, HN2, and HN3—alkylated the phosphocholine headgroup of POPC, a ubiquitous lipid found in virtually all eukaryotic cells. The corresponding hydrolysis products of the selected mustard agents did not react with POPC. Additionally, Lewisites L1 and L2, as well as their respective hydrolysis products, were unreactive. Utilising HILIC-MS/MS, we were able to demonstrate the presence of alkylated PCs in plasma samples spiked with trace levels of HD. This suggests that mustard-alkylated PCs could function as biomarkers of mustard agent exposure. To the best of our knowledge, this is the first time mustard adducts of PCs have been reported. The possible contribution of lipid adduction to the overall pathophysiology of mustard agents is an interesting topic for future studies.

## Supplementary Information

Below is the link to the electronic supplementary material.

Supplementary file1 SI A: HRMS fragmentation patterns (DOCX 617 KB)

Supplementary file2 SI B: Plasma exposure experiments (DOCX 1.50 MB)

## Data Availability

The datasets generated and analysed during the current study are available from the corresponding author upon reasonable request.

## Abbreviations

(H)ESI: (Heated) electrospray ionisation
(HR)MS: (High-resolution) mass spectrometry
ACN: Acetonitrile
AmF: Ammonium formate
b-CVAOA: Bis(2-chlorovinyl)arsinic acid
Cer: Ceramide
CVAOA: 2-Chlorovinylarsonic acid
CWA: Chemical warfare agent
DCM: Dichloromethane
DG: Diglycerides
EDEA: Bis(ethanol)ethylamine
EDEA-POPC: POPC adduct of hemi-HN1
FA: Formic acid
GC: Gas chromatography
HCD: High-energy collisional dissociation
HD: Sulphur mustard; bis(2-chloroethyl)sulphide
HD-PC: HD-alkylated PC
HD-POPC: POPC adduct of HD
hemi-HN3-POPC: POPC adduct of hemi-HN3
HILIC: Hydrophilic interaction liquid chromatography
HN1: Nitrogen mustard 1; bis(2-chloroethyl)ethylamine
HN1-POPC: POPC adduct of HN1
HN2: Nitrogen mustard 2; bis(2-chloroethyl)methylamine
HN2-POPC: POPC adduct of HN2
HN3: Nitrogen mustard 3; tris(2-chloroethyl)amine
HN3-POPC: POPC adduct of HN3
HPLC: High-performance liquid chromatography
IPA: 2-Propanol
L1: Lewisite 1; 2-chlorovinylarsinedichloride
L2: Lewisite 2; bis(2-chlorovinyl)arsine chloride
LC: (Ultra-high-performance) liquid chromatography
*m*/*z*: Mass-to-charge ratio
MDEA: Bis(ethanol)methylamine
MDEA-POPC: POPC adduct of hemi-HN2
MeOH: Methanol
MRM: Multi-reaction monitoring
MS/(HR)MS: (High-resolution) tandem mass spectrometry
PC: Phosphatidylcholine
PE: Phosphatidylethanolamine
POPC: 1-Palmitoyl-2-oleoyl-glycero-*sn*-3-phosphocholine
Q: Sesquimustard; 1,2-bis((2-chloroethyl)thio)ethane
Q-diol: 1,2-Bis(2-hydroxyethylthio)ethane
Q-ol-POPC: POPC adduct of hemi-Q
Q-POPC: POPC adduct of Q
SM: Sphingomyelin
TDG: Thiodiglycol
TDG-POPC: POPC adduct of hemi-HD
TEA: Tris(ethanol)amine
TEA-POPC: POPC adduct of hemi-HN3
TG: Triglycerides
