# Supplementary material for: Identification of adducts formed between phosphatidylcholine and mustard agents
Source: Anal Bioanal Chem. 2026 Jun 13;418(15):4951–66. doi: 10.1007/s00216-026-06600-4 (PMC13388424; doi:10.1007/s00216-026-06600-4)
Supplement: Supplementary file 1 — Supplementary file1 SI A: HRMS fragmentation patterns (DOCX 617 KB) [file 216_2026_6600_MOESM1_ESM.docx]

**Supplementary information A for**

**Identification of adducts formed between phosphatidylcholine and mustard agents**

Table of Contents

[1 Fragmentation patterns of identified analytes 2](#_Toc223175468)

Fig. A.1. Structure of the POPC-substituent 3

Table A.1. LC-MS/HRMS fragmentation pattern of HD-POPC 3

Table A.2. LC-MS/MS/HRMS fragmentation pattern of HD-POPC. Isolated ion = *m*/*z* 306.06902 5

Table A.3. LC-MS/MS/HRMS fragmentation pattern of HD-POPC. Isolated ion = *m*/*z* 278.03772 6

Table A.4. LC-MS/MS/HRMS fragmentation pattern of HD-POPC. Isolated ion = *m*/*z* 242.06104 7

Table A.5. LC-MS/HRMS fragmentation pattern of TDG-POPC 8

Table A.6. LC-MS/HRMS fragmentation pattern of Q-POPC 10

Table A.7. LC-MS/HRMS fragmentation pattern of Q-ol-POPC 12

Table A.8. LC-MS/HRMS fragmentation pattern of HN1-POPC 14

Table A.9. LC-MS/HRMS fragmentation pattern of EDEA-POPC 16

Table A.10. LC-MS/HRMS fragmentation pattern of HN2-POPC 17

Table A.11. LC-MS/HRMS fragmentation pattern of MDEA-POPC 19

Table A.12. LC-MS/HRMS fragmentation pattern of HN3-POPC 21

Table A.13. LC-MS/HRMS fragmentation pattern of Hemi-HN3-POPC 23

Table A.14. LC-MS/HRMS fragmentation pattern of TEA-POPC 25

# Fragmentation patterns of identified analytes


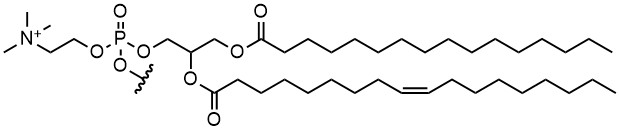


Fig. A.1. Structure of the POPC-substituent. All mustard agents alkylated the hydroxyl-group in the phosphocholine headgroup of POPC. In Table 2 the POPC-substituent in the complexes is abbreviated as POPC.

Table A.1. LC-MS/HRMS fragmentation pattern of HD-POPC. Mass difference is presented as reported by the instrument software.

| **HD-POPC** | | **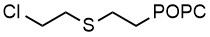** | | |
| --- | --- | --- | --- | --- |
|   577.51907 | | | | |
| **Predicted formula** | **Proposed structure** | **Theoretical *m*/*z*** | **Measured *m*/*z*** | **Mass difference [ppm]** |
| C_46_H_90_ClNO_8_PS^+^ | 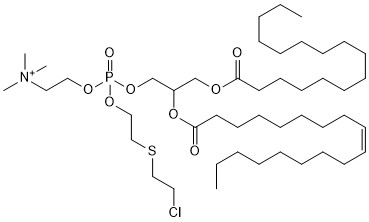 | 882.58078 | 882.58095 | 0.19025 |
| C_37_H_69_O_4_^+^ | 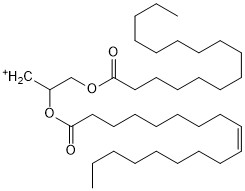 | 577.51904 | 577.51907 | 0.05058 |
| C_9_H_22_ClNO_4_PS^+^ | 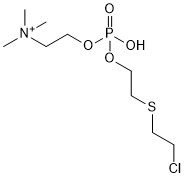 | 306.06902 | 306.06919 | 0.54646 |
| C_7_H_18_ClNO_4_PS^+^ | 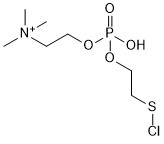 | 278.03772 | 278.03776 | 0.14171 |
| C_7_H_17_NO_4_PS^+^ | 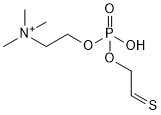 | 242.06104 | 242.06104 | -0.00770 |
| C_5_H_15_NO_4_P^+^ | 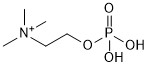 | 184.07332 | 184.07333 | 0.03688 |
| C_4_H_8_ClS^+^ | 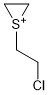 | 123.00298 | 123.00294 | -0.28110 |
| C_5_H_12_N^+^ | 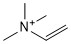 | 86.09643 | 86.09641 | -0.15227 |

Table A.2. LC-MS/MS/HRMS fragmentation pattern of HD-POPC. Isolated ion = *m*/*z* 306.06902. Mass difference is presented as reported by the instrument software.

| **HD-POPC** | | **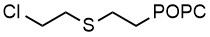** | | | |
| --- | --- | --- | --- | --- | --- |
| **** | | | | | |
| **Predicted formula** | **Proposed structure** | | **Theoretical *m*/*z*** | **Measured *m*/*z*** | **Mass difference [ppm]** |
| C_9_H_22_ClNO_4_PS^+^ | 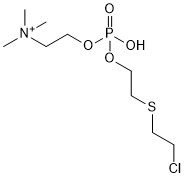 | | 306.06902 | 306.06898 | -012476 |
| C_7_H_18_ClNO_4_PS^+^ | 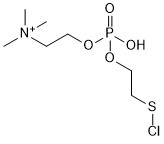 | | 278.03772 | 278.03773 | 0.3297 |
| C_7_H_17_NO_4_PS^+^ | 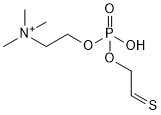 | | 242.06104 | 242.06102 | -0.10382 |
| C_5_H_15_NO_4_P^+^ | 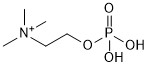 | | 184.07332 | 184.07335 | 0.17229 |
| C_4_H_8_ClS^+^ | 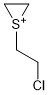 | | 123.00298 | 123.00299 | 0.15486 |
| C_5_H_14_NO^+^ | 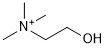 | | 104.10699 | 104.10692 | -0.63697 |
| C_5_H_12_N^+^ | 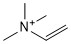 | | 86.09643 | 86.09638 | -0.50543 |

Table A.3. LC-MS/MS/HRMS fragmentation pattern of HD-POPC. Isolated ion = *m*/*z* 278.03772. Mass difference is presented as reported by the instrument software.

| **HD-POPC** | | **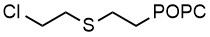** | | | |
| --- | --- | --- | --- | --- | --- |
| **** | | | | | |
| **Predicted formula** | **Proposed structure** | | **Theoretical *m*/*z*** | **Measured *m*/*z*** | **Mass difference [ppm]** |
| C_7_H_18_ClNO_4_PS^+^ | 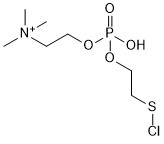 | | 278.03772 | 278.03777 | 0.19742 |
| C_5_H_15_NO_4_P^+^ | 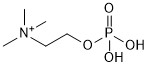 | | 184.07332 | 184.07331 | -0.07525 |
| C_5_H_12_N^+^ | 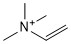 | | 86.09643 | 86.09637 | -0.67074 |

Table A.4. LC-MS/MS/HRMS fragmentation pattern of HD-POPC. Isolated ion = *m*/*z* 242.06104. Mass difference is presented as reported by the instrument software.

| **HD-POPC** | | **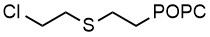** | | | |
| --- | --- | --- | --- | --- | --- |
|   184.07326 | | | | | |
| **Predicted formula** | **Proposed structure** | | **Theoretical *m*/*z*** | **Measured *m*/*z*** | **Mass difference [ppm]** |
| C_7_H_17_NO_4_PS^+^ | 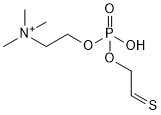 | | 242.06104 | 242.06102 | -0.07541 |
| C_5_H_15_NO_4_P^+^ | 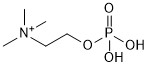 | | 184.07332 | 184.07326 | -0.31609 |
| C_4_H_8_O_4_PS^+^ | 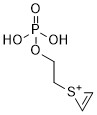 | | 182.98754 | 182.98751 | -0.18554 |
| C_5_H_12_N^+^ | 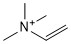 | | 86.09643 | 86.09639 | -0.37075 |

Table A.5. LC-MS/HRMS fragmentation pattern of TDG-POPC. Mass difference is presented as reported by the instrument software.

| **TDG-POPC** | | **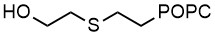** | | |
| --- | --- | --- | --- | --- |
|   565.52015 | | | | |
| **Predicted formula** | **Proposed structure** | **Theoretical *m*/*z*** | **Measured *m*/*z*** | **Mass difference [ppm]** |
| C_46_H_91_NO_9_PS^+^ | 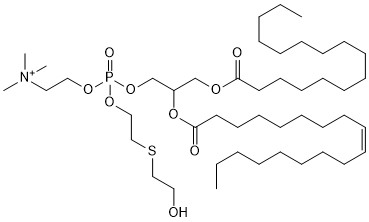 | 864.61467 | 864.61473 | 0.06887 |
| C_36_H_69_O_4_^+^ | 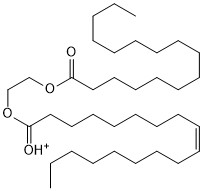 | 565.51904 | 565.52015 | 1.96644 |
| C_9_H_23_NO_5_PS^+^ | 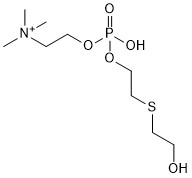 | 288.10291 | 288.10293 | 0.07661 |
| C_7_H_19_NO_5_PS^+^ | 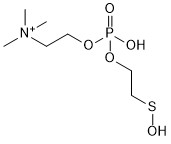 | 260.07161 | 260.07162 | 0.06436 |
| C_7_H_17_NO_4_PS^+^ | 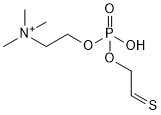 | 242.06104 | 242.06102 | -0.07674 |
| C_5_H_15_NO_4_P^+^ | 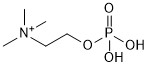 | 184.07332 | 184.07331 | -0.05780 |
| C_4_H_9_OS^+^ | 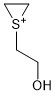 | 105.03866 | 105.03685 | -0.09398 |
| C_5_H_12_N^+^ | 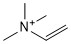 | 86.09643 | 86.09642 | -0.05754 |

Table A.6. LC-MS/HRMS fragmentation pattern of Q-POPC. Mass difference is presented as reported by the instrument software.

| **Q-POPC** | | **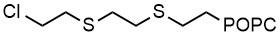** | | |
| --- | --- | --- | --- | --- |
|   154.97502  338.04108  577.51902  306.06903  183.00635  86.09643 | | | | |
| **Predicted formula** | **Proposed structure** | **Theoretical *m*/*z*** | **Measured *m*/*z*** | **Mass difference [ppm]** |
| C_48_H_94_ClNO_8_PS_2_^+^ | 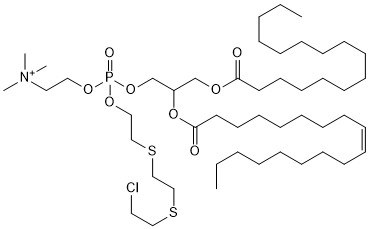 | 942.58415 | 942.58450 | 0.36993 |
| C_37_H_69_O_4_^+^ | 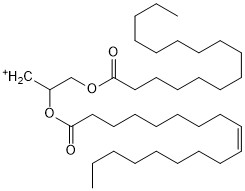 | 577.51904 | 577.51902 | -0.02999 |
| C_11_H_26_ClNO_4_PS_2_^+^ | 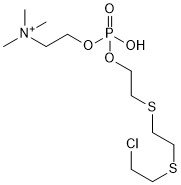 | 366.07239 | 366.07257 | 0.48741 |
| C_9_H_22_ClNO_4_PS_2_^+^ | 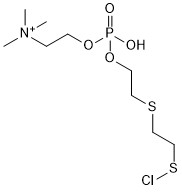 | 338.04109 | 338.04108 | -0.03387 |
| C_11_H_25_NO_4_PS_2_^+^ | 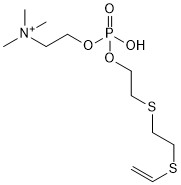 | 330.09571 | 330.09580 | 0.25720 |
| C_9_H_22_ClNO_4_PS^+^ | 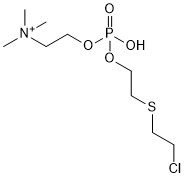 | 306.06902 | 306. 06903 | 0.01932 |
| C_7_H_18_ClNO_4_PS^+^ | 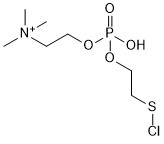 | 278.03772 | 278.03771 | -0.02470 |
| C_7_H_17_NO_4_PS^+^ | 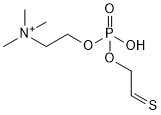 | 242.06104 | 242.06111 | 0.28875 |
| C_5_H_15_NO_4_P^+^ | 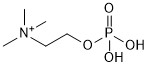 | 184-07332 | 184.07334 | 0.09445 |
| C_6_H_12_ClS_2_^+^ | 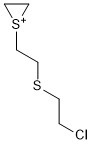 | 183.00635 | 183.00635 | 0.04561 |
| C_4_H_8_ClS_2_^+^ | 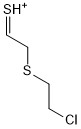 | 154.97505 | 154.97502 | -015542 |
| C_4_H_8_ClS^+^ | 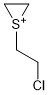 | 123.00298 | 123.00294 | -0.28318 |
| C_5_H_12_N^+^ | 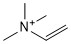 | 86.09643 | 86.09641 | -0.21238 |

Table A.7. LC-MS/HRMS fragmentation pattern of Q-ol-POPC. Mass difference is presented as reported by the instrument software.

| **Q-ol-POPC** | | **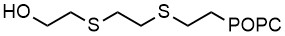** | | |
| --- | --- | --- | --- | --- |
|   577.51881  320.07501  86.09641  137.00895  260.07162  330.09573 | | | | |
| **Predicted formula** | **Proposed structure** | **Theoretical *m*/*z*** | **Measured *m*/*z*** | **Mass difference [ppm]** |
| C_48_H_95_NO_9_PS_2_^+^ | 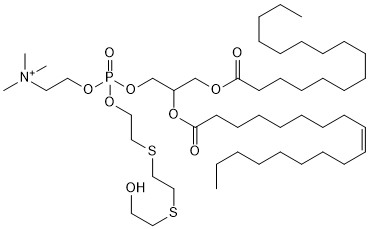 | 924.61804 | 924.61843 | 0.42033 |
| C_37_H_69_O_4_^+^ | 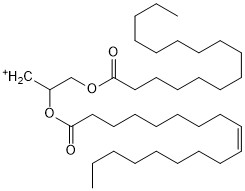 | 577.51904 | 577.51881 | -0.40066 |
| C_11_H_27_NO_5_PS_2_^+^ | 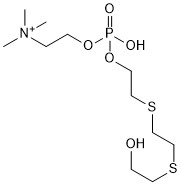 | 348.10628 | 348.10650 | 0.14181 |
| C_11_H_25_NO_4_PS_2_^+^ | 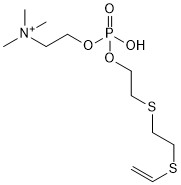 | 330.09571 | 330.09573 | 0.03790 |
| C_9_H_23_NO_5_PS_2_^+^ | 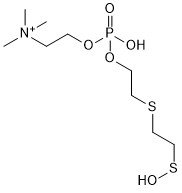 | 320.07498 | 320.07501 | 0.10650 |
| C_9_H_23_NO_5_PS^+^ | 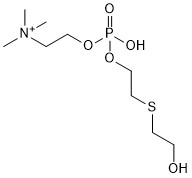 | 288.10291 | 288.10294 | 0.10392 |
| C_7_H_19_NO_5_PS^+^ | 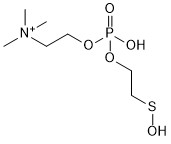 | 260.07161 | 260.07162 | 0.10392 |
| C_7_H_17_NO_4_PS^+^ | 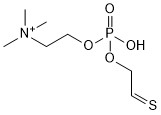 | 242.06104 | 242.06102 | -0.07268 |
| C_5_H_15_NO_4_P^+^ | 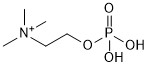 | 184.07332 | 184.07334 | 0.12578 |
| C_6_H_13_OS_2_^+^ | 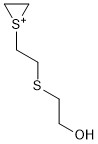 | 165.04023 | 165.04028 | 0.30312 |
| C_4_H_9_OS_2_^+^ | 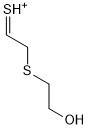 | 137.00893 | 137.00895 | 0.09893 |
| C_4_H_9_OS^+^ | 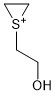 | 105.03686 | 105.03685 | -0.13920 |
| C_5_H_12_N^+^ | 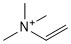 | 86.09643 | 86.09641 | -0.20772 |

Table A.8. LC-MS/HRMS fragmentation pattern of HN1-POPC. Mass difference is presented as reported by the instrument software.

| **HN1-POPC** | | **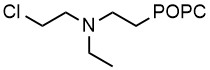** | | |
| --- | --- | --- | --- | --- |
|   222.08897  184.07329  281.16244 | | | | |
| **Predicted formula** | **Proposed structure** | **Theoretical *m*/*z*** | **Measured *m*/*z*** | **Mass difference [ppm]** |
| C_48_H_95_ClN_2_O_8_P^+^ | 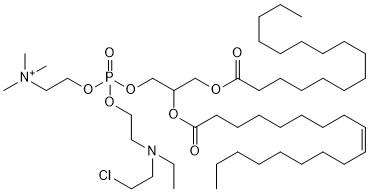 | 893.65091 | 893.66070 | -0.23673 |
| C_38_H_67_ClO_6_P^+^ | 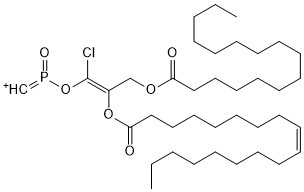 | 685.43583 | 685.43551 | -0.46061 |
| C_11_H_27_ClN_2_O_4_P^+^ | 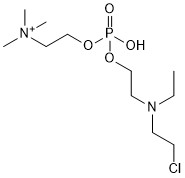 | 317.13915 | 317.13915 | 0.02186 |
| C_11_H_26_N_2_O_4_P^+^ | 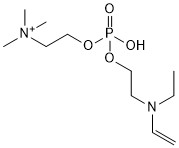 | 281.16247 | 281.16244 | -0.10629 |
| C_8_H_18_ClNO_4_P^+^ | 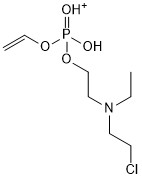 | 258.06565 | 258.06558 | -0.24783 |
| C_8_H_17_NO_4_P^+^ | 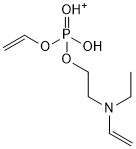 | 222.08897 | 222.08897 | -0.02048 |
| C_5_H_15_NO_4_P^+^ | 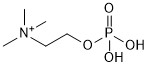 | 184.07332 | 184.07329 | -0.15521 |
| C_6_H_13_ClN^+^ | 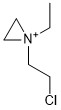 | 134.07310 | 134.07309 | -0.12412 |
| C_5_H_12_N^+^ | 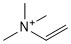 | 86.09643 | 86.09644 | 0.15702 |

Table A.9. LC-MS/HRMS fragmentation pattern of EDEA-POPC. Mass difference is presented as reported by the instrument software.

| **EDEA-POPC** | | **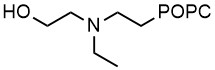** | | |
| --- | --- | --- | --- | --- |
|   86.09642  184.07329 | | | | |
| **Predicted formula** | **Proposed structure** | **Theoretical *m*/*z*** | **Measured *m*/*z*** | **Mass difference [ppm]** |
| C_48_H_96_N_2_O_9_P^+^ | 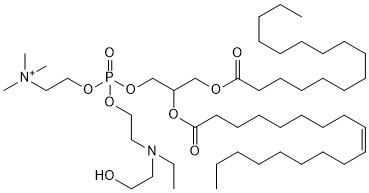 | 875.68480 | 875.68461 | -0.21417 |
| C_11_H_28_N_2_O_5_P^+^ | 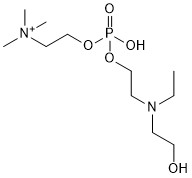 | 299.17303 | 299.17301 | -0.09618 |
| C_8_H_19_NO_5_P^+^ | 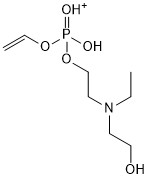 | 240.09954 | 240.09953 | -0.03514 |
| C_5_H_15_NO_4_P^+^ | 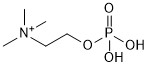 | 184.07332 | 184.07329 | -0.14649 |
| C_6_H_14_NO^+^ | 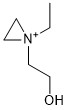 | 116.10699 | 116.10699 | 0.02206 |
| C_5_H_12_N^+^ | 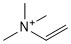 | 86.09643 | 86.09642 | -0.02470 |

Table A.10. LC-MS/HRMS fragmentation pattern of HN2-POPC. Mass difference is presented as reported by the instrument software.

| **HN2-POPC** | | **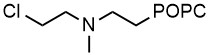** | | |
| --- | --- | --- | --- | --- |
|   86.09641  208.07332  267.14687  577.51894 | | | | |
| **Predicted formula** | **Proposed structure** | **Theoretical *m*/*z*** | **Measured *m*/*z*** | **Mass difference [ppm]** |
| C_47_H_93_ClN_2_O_8_P^+^ | 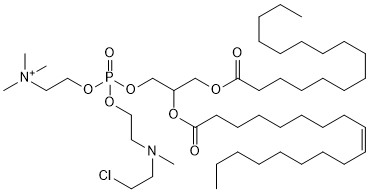 | 879.63526 | 879.63535 | -0.10012 |
| C_37_H_69_O_4_^+^ | 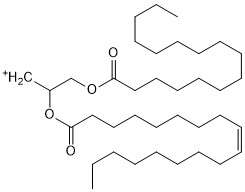 | 577.51904 | 577.51894 | -0.17005 |
| C_10_H_25_ClN_2_O_4_P^+^ | 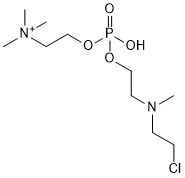 | 303.12350 | 303.12357 | 0.24107 |
| C_7_H_16_ClNO_4_P^+^ | 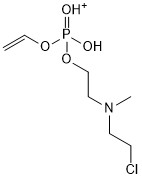 | 267.14682 | 267.14683 | 0.05525 |
| C_10_H_24_N_2_O_4_P^+^ | 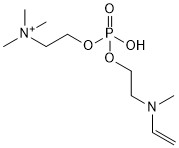 | 244.05000 | 244.05000 | 0.01047 |
| C_7_H_15_NO_4_P^+^ | 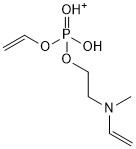 | 208.07332 | 208.07332 | -0.01164 |
| C_5_H_15_NO_4_P^+^ | 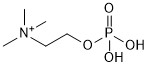 | 184.07332 | 184.07333 | 0.03575 |
| C_5_H_11_N^+^ | 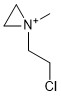 | 120.05745 | 120.05746 | 0.04286 |
| C_5_H_12_N^+^ | 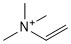 | 86.09643 | 86.09641 | -0.19188 |

Table A.11. LC-MS/HRMS fragmentation pattern of MDEA-POPC. Mass difference is presented as reported by the instrument software.

| **MDEA-POPC** | | **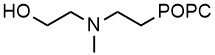** | | |
| --- | --- | --- | --- | --- |
|   577.51908  86.09641 | | | | |
| **Predicted formula** | **Proposed structure** | **Theoretical *m*/*z*** | **Measured *m*/*z*** | **Mass difference [ppm]** |
| C_47_H_94_N_2_O_9_P^+^ | 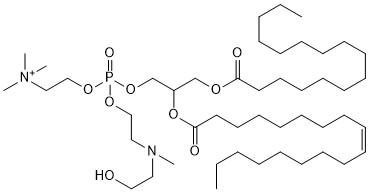 | 861.66915 | 861.66906 | -0.09894 |
| C_37_H_69_O_4_^+^ | 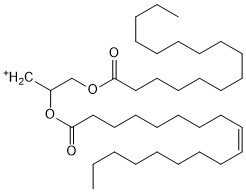 | 577.51904 | 577.51908 | 0.08187 |
| C_10_H_26_N_2_O_5_P^+^ | 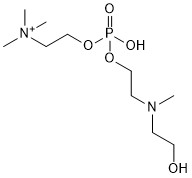 | 285.15738 | 285.15740 | 0.05096 |
| C_7_H_17_NO_5_P^+^ | 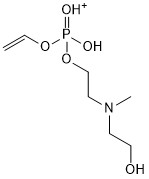 | 226.08389 | 226.08388 | -0.03657 |
| C_5_H_15_NO_4_P^+^ | 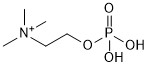 | 184.07332 | 184.07331 | -0.06348 |
| C_5_H_12_NO^+^ | 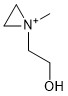 | 102.09134 | 102.09133 | -0.08252 |
| C_5_H_12_N^+^ | 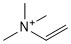 | 86.09643 | 86.09641 | -0.18395 |

Table A.12. LC-MS/HRMS fragmentation pattern of HN3-POPC. Mass difference is presented as reported by the instrument software.

| **HN3-POPC** | | **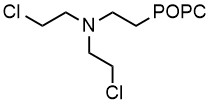** | | |
| --- | --- | --- | --- | --- |
| 292.02667 | | | | |
| **Predicted formula** | **Proposed structure** | **Theoretical *m*/*z*** | **Measured *m*/*z*** | **Mass difference [ppm]** |
| C_48_H_94_Cl_2_N_2_O_8_P^+^ |  | 927.61194 | 927.61172 | -0.22899 |
| C_11_H_26_Cl_2_N_2_O_4_P^+^ |  | 351.10018 | 351.10017 | -0.02335 |
| C_11_H_25_ClN_2_O_4_P^+^ |  | 315.12350 | 315.12351 | 0.03509 |
| C_8_H_17_Cl_2_NO_4_P^+^ |  | 292.02668 | 292.02667 | -0.00652 |
| C_8_H_16_ClNO_4_P^+^ |  | 256.05000 | 256.04999 | -0.02512 |
| C_5_H_15_NO_4_P^+^ |  | 184.07332 | 184.07331 | -0.07644 |
| C_6_H_12_Cl_2_N^+^ |  | 168.03413 | 168.03414 | 0.06517 |
| C_5_H_12_N^+^ |  | 86.09643 | 86.09641 | -0.20199 |

Table A.13. LC-MS/HRMS fragmentation pattern of Hemi-HN3-POPC. Mass difference is presented as reported by the instrument software.

| **Hemi-HN3-POPC** | |  | | |
| --- | --- | --- | --- | --- |
| 297.157371 | | | | |
| **Predicted formula** | **Proposed structure** | **Theoretical *m*/*z*** | **Measured *m*/*z*** | **Mass difference [ppm]** |
| C_48_H_95_ClN_2_O_9_P^+^ |  | 909.64582 | 909.64558 | -0.26625 |
| C_11_H_27_ClN_2_O_5_P^+^ |  | 333.13406 | 333.13406 | -0.00298 |
| C_11_H_26_N_2_O_5_P^+^ |  | 297.15738 | 297.15737 | -0.05869 |
| C_8_H_18_ClN_2_O_5_P^+^ |  | 274.06056 | 274.06060 | 0.12584 |
| C_8_H_17_NO_5_P^+^ |  | 238.08389 | 238.08386 | -0.10143 |
| C_5_H_15_NO_4_P^+^ |  | 184.07332 | 184.07331 | -0.03533 |
| C_6_H_13_ClNO^+^ |  | 150.06802 | 150.06800 | -0.09273 |
| C_5_H_12_N^+^ |  | 86.09643 | 86.09641 | -0.15023 |

Table A.14. LC-MS/HRMS fragmentation pattern of TEA-POPC. Mass difference is presented as reported by the instrument software.

| **TEA-POPC** | |  | | |
| --- | --- | --- | --- | --- |
| 184.073331 | | | | |
| **Predicted formula** | **Proposed structure** | **Theoretical *m*/*z*** | **Measured *m*/*z*** | **Mass difference [ppm]** |
| C_48_H_96_N_2_O_10_P^+^ |  | 891.67971 | 891.67941 | -0.33842 |
| C_11_H_28_N_2_O_6_P^+^ |  | 315.16795 | 315.16797 | 0.06856 |
| C_8_H_19_NO_6_P^+^ |  | 256.09445 | 256.09444 | -0.02943 |
| C_5_H_15_NO_4_P^+^ |  | 184.07332 | 184.07331 | -0.08254 |
| C_6_H_14_NO_2_^+^ |  | 132.10191 | 132.10191 | 0.03112 |
| C_5_H_12_N^+^ |  | 86.09643 | 86.09643 | 0.08095 |
