# Supplementary material for: Identification of adducts formed between phosphatidylcholine and mustard agents
Source: Anal Bioanal Chem. 2026 Jun 13;418(15):4951–66. doi: 10.1007/s00216-026-06600-4 (PMC13388424; doi:10.1007/s00216-026-06600-4)
Supplement: Supplementary file 2 — Supplementary file2 SI B: Plasma exposure experiments (DOCX 1.50 MB) [file 216_2026_6600_MOESM2_ESM.docx]

**Supplementary information B for**

**Identification of adducts formed between phosphatidylcholine and mustard agents**

**Table of Contents**

[1 Method parameters 2](#_Toc229579258)

[Table B.1. Method settings of the MRM-method. 2](#_Toc229579324)

[2 Chromatograms and spectra of plasma sample 2](#_Toc229579259)

[Fig. B.1. Par184 TIC of plasma lipids.. 2](#_Toc229579171)

[Fig. B.2. MRM chromatograms of HD-PC-34:1 (HD-POPC and its isomers) and HD-PC-34:2, from analysed plasma lipid-extract samples, as well as a prepared HD-POPC reference sample for comparison.. 5](#_Toc229579172)

[Fig. B.3. LC-MS/HRMS spectra obtained by HCD fragmentation of the monoisotopic peak ([M+H]^+^) and the +1 and +2 isotopic peaks ([M+1+H]^+^ and [M+2+H]^+^, respectively) of HD-POPC.. 6](#_Toc229579173)

[Fig. B.4. LC-HRMS TIC (top) measured for a plasma sample exposed to 250 ppm of HD. The EICs, extracted with 2.5 ppm accuracy, of HD-PC34:2 (RT 3.27 min) and HD-POPC (3.23 min) are presented in the figures in the middle and at the bottom, respectively. 7](#_Toc229579174)

[Fig. B.5. LC‒MS/HRMS spectra measured for HD-exposed plasma samples.. 8](#_Toc229579175)

# Method parameters

Table B.1. Method settings of the MRM-method.

| **HD-PC 34:1**  **Transitions** | **Collision energy (V)** | **Cone voltage (V)** |
| --- | --- | --- |
| 882.6 > 306 | 35 | 20 |
| 882.6 > 278 | 45 | 20 |
| 882.6 > 242 | 45 | 20 |
| **HD-PC 34:2**  **Transitions** | **Collision energy (V)** | **Cone voltage (V)** |
| 880.6 > 306 | 35 | 20 |
| 880.6 > 278 | 45 | 20 |
| 880.6 > 242 | 45 | 20 |
| **POPC**  **Transition** | **Collision energy (V)** | **Cone voltage (V)** |
| 760.6 > 184 | 8* | 20 |

*Sub-optimal collision energy was chosen to avoid signal saturation, see main text.

# Chromatograms and spectra of plasma sample


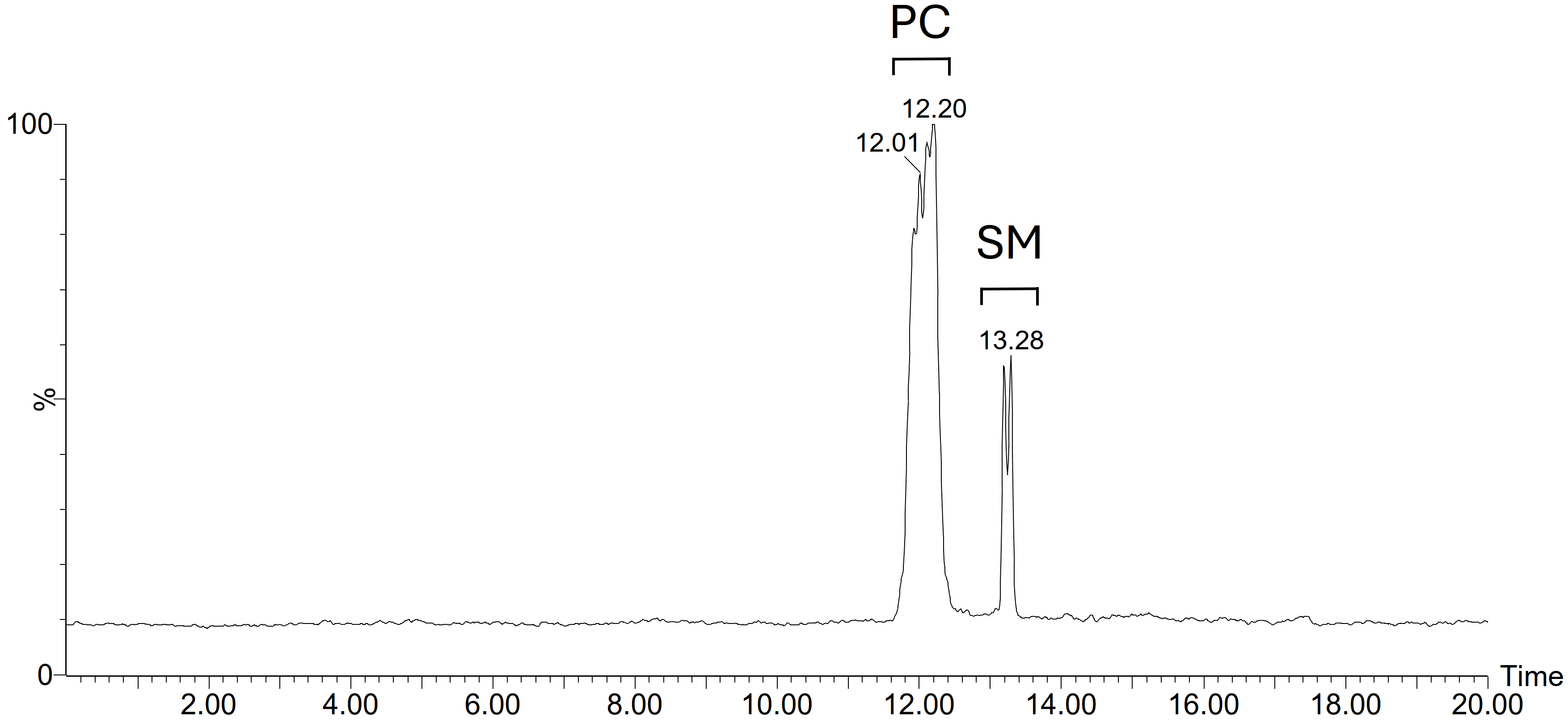


Fig. B.1. Par184 TIC of plasma lipids. The spectra presented in Fig. 7 in the main text is extracted from the PC peak at RT 11.50–12-60 min.

| **A** | **HD-PC-34:1 (HD-POPC and its isomers) – BLANK:**  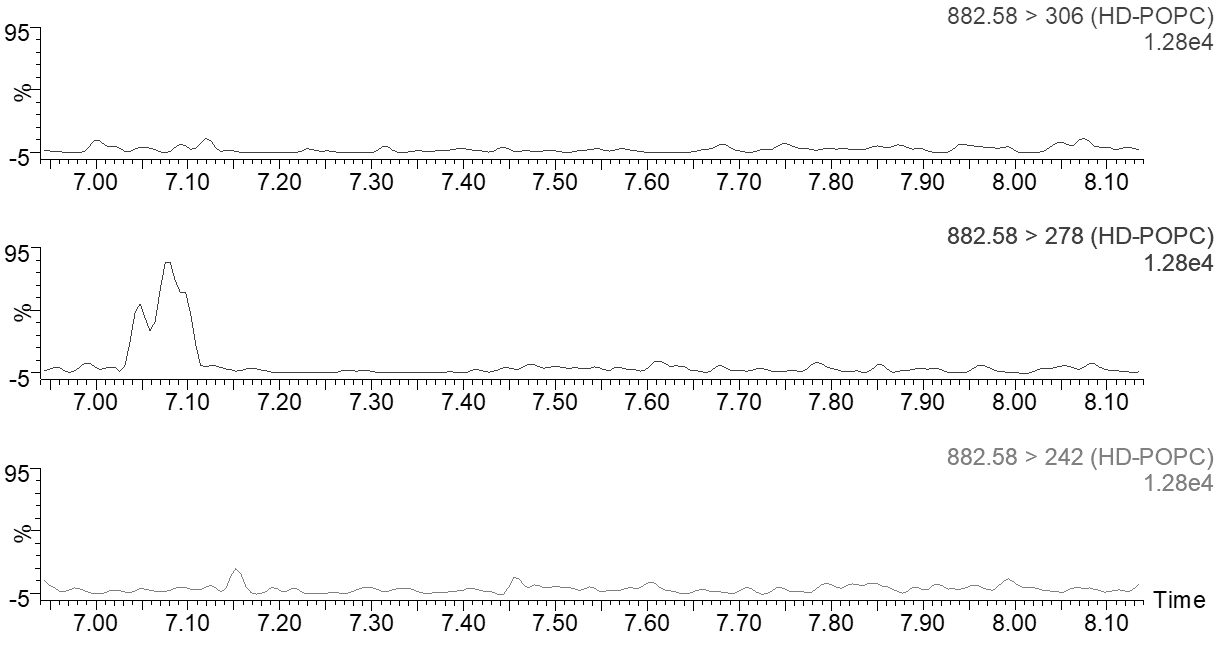  Q  q 1  q 2 |
| --- | --- |
| **B** | **HD-PC-34:1 (HD-POPC and its isomers) – SAMPLE:**  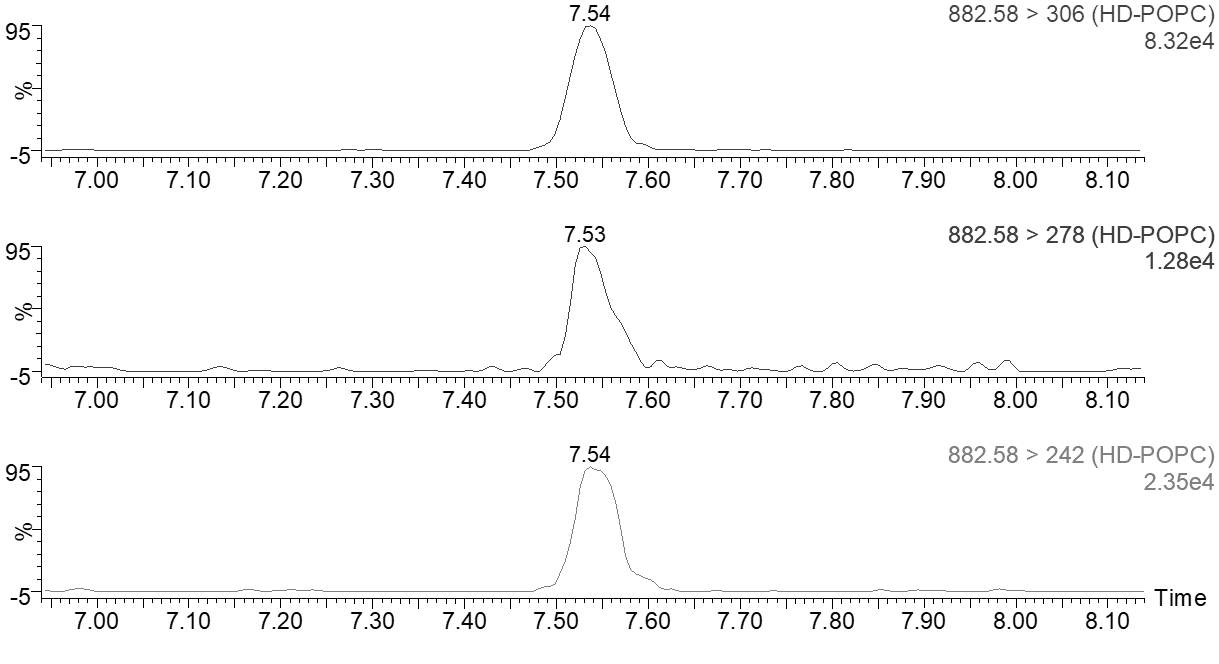  Q  q 1  q 2 |
| **C** | **HD-PC-34:2 – BLANK:**  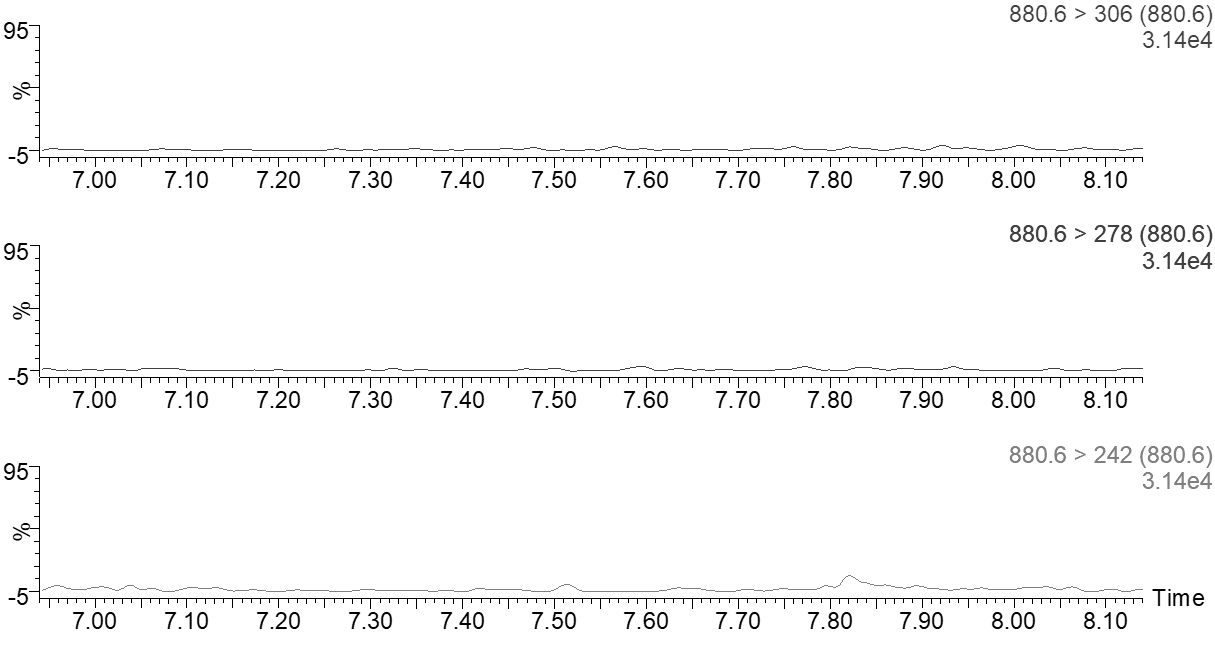  q 2  q 1  Q |
| **D** | **HD-PC-34:2 – SAMPLE:**  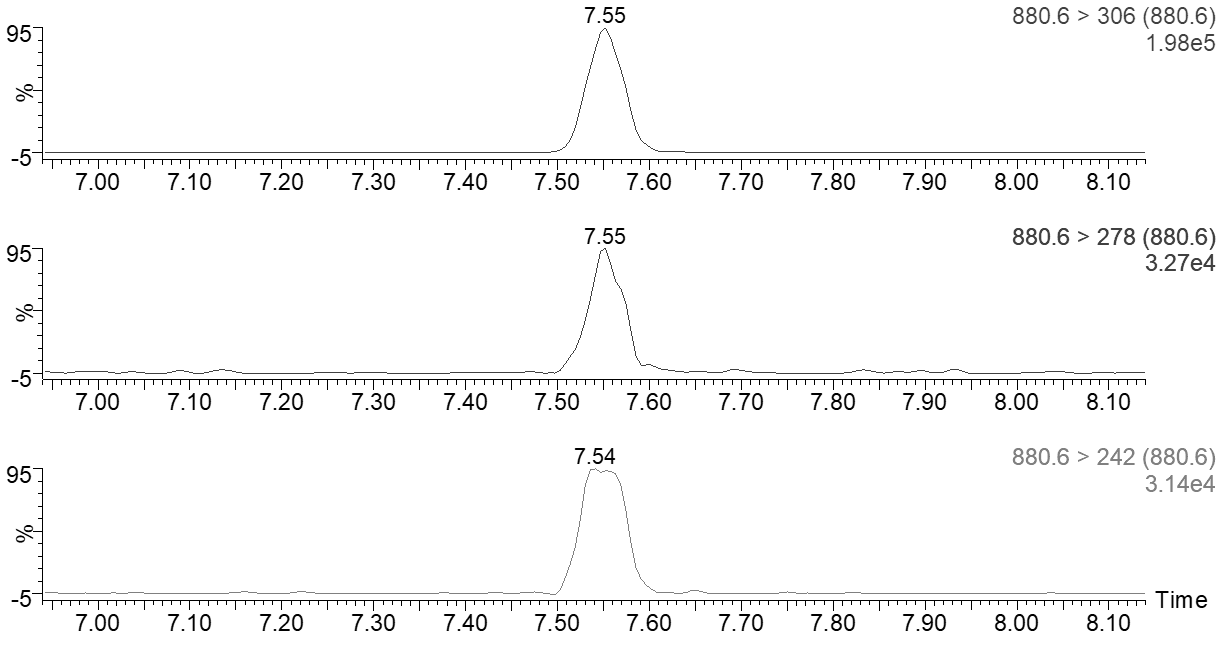  q 2  q 1  Q |
| **E** | **HD-POPC – REFERENCE:**  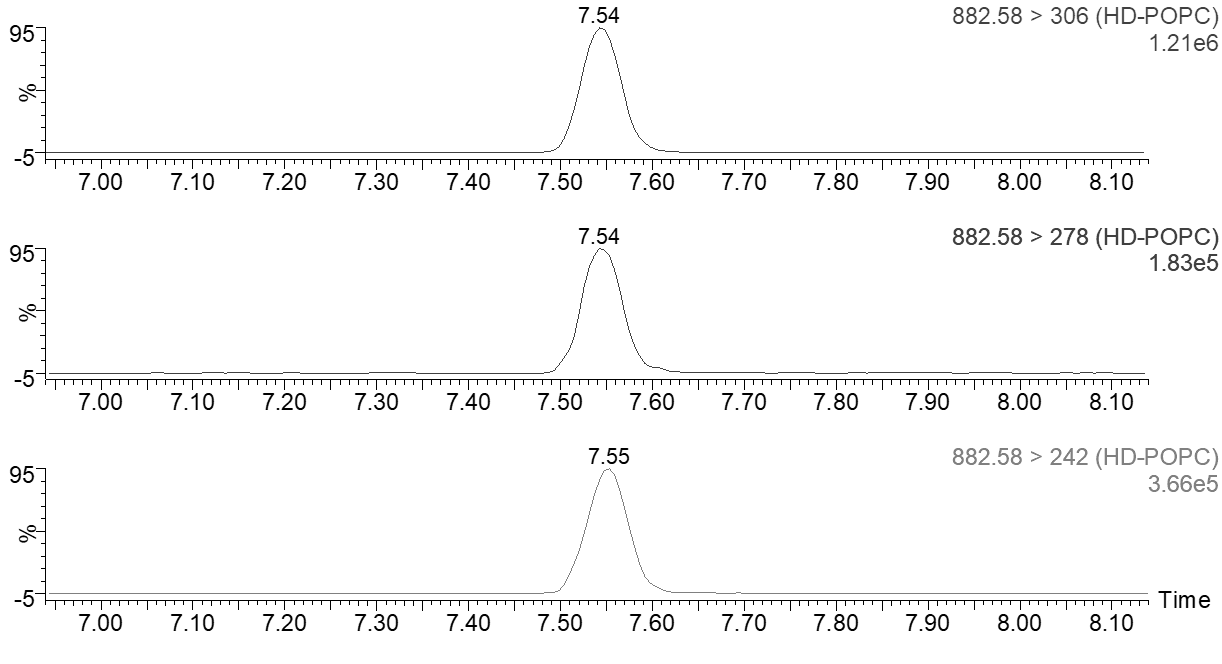  q 2  q 1  Q |
|  | Fig. B.2. MRM chromatograms of HD-PC-34:1 (HD-POPC and its isomers) and HD-PC-34:2, from analysed plasma lipid-extract samples, as well as a prepared HD-POPC reference sample for comparison. The transitions (Q, q1 and q2) are visible in the chromatogram text and are also describe in Table 1 of this document.  A: HD-PC-34:1 signal in blank (unspiked) plasma sample.  B: HD-PC-34:1 signal in HD-spiked plasma sample.  C: HD-PC-34:2 signal in blank (unspiked) plasma sample.  D: HD-PC-34:2 signal in HD-spiked plasma sample.  E: HD-PC-34:1 signal in HD-POPC reference sample. |


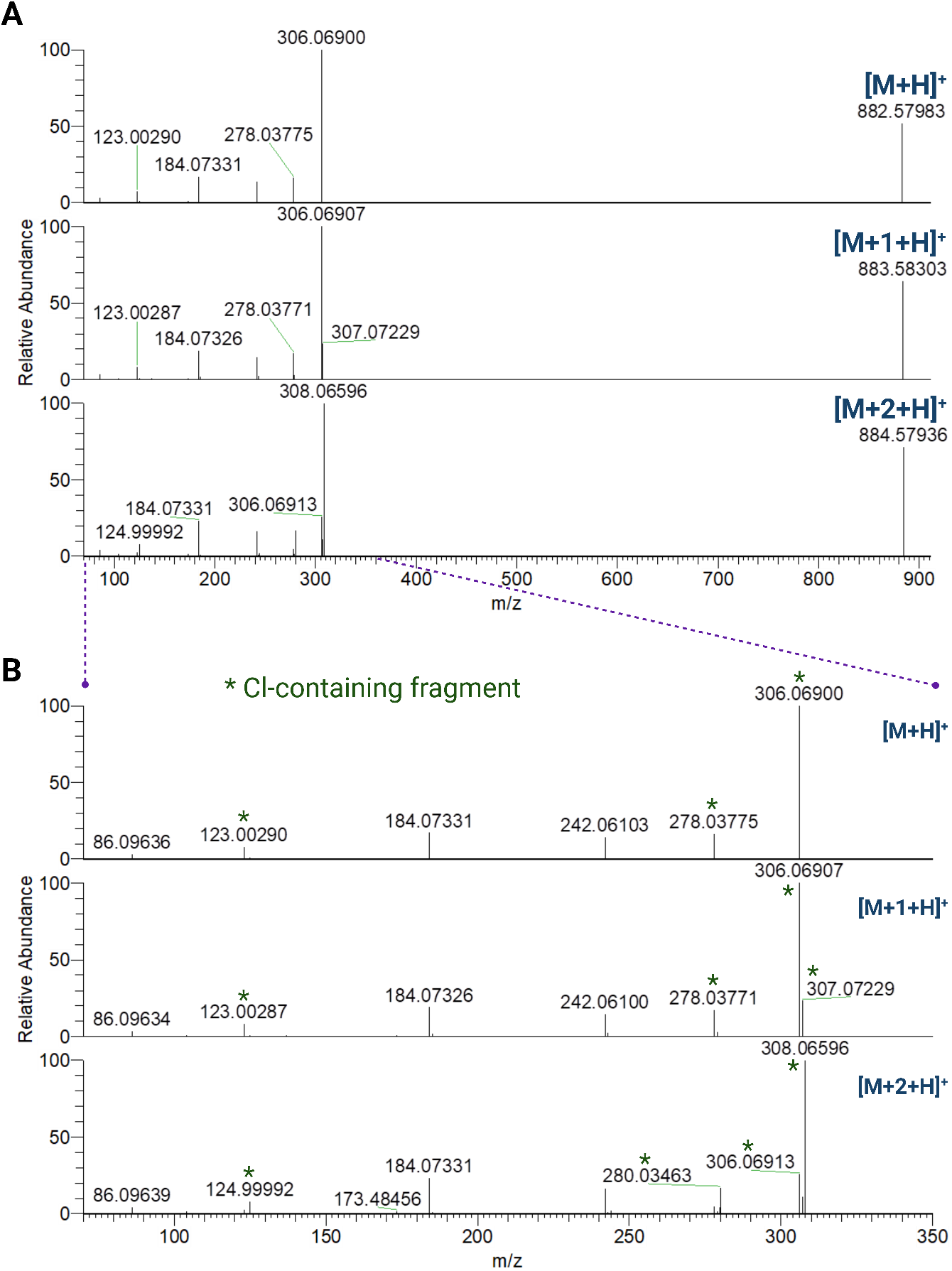


Fig. B.3. LC-MS/HRMS spectra obtained by HCD fragmentation of the monoisotopic peak ([M+H]^+^) and the +1 and +2 isotopic peaks ([M+1+H]^+^ and [M+2+H]^+^, respectively) of HD-POPC. Spectra of the whole measured *m*/*z* range (70‒902) are presented in panel A. A close up of the *m*/*z* range 70‒350 with chlorine-containing fragments marked with asterixis, is presented in panel B.


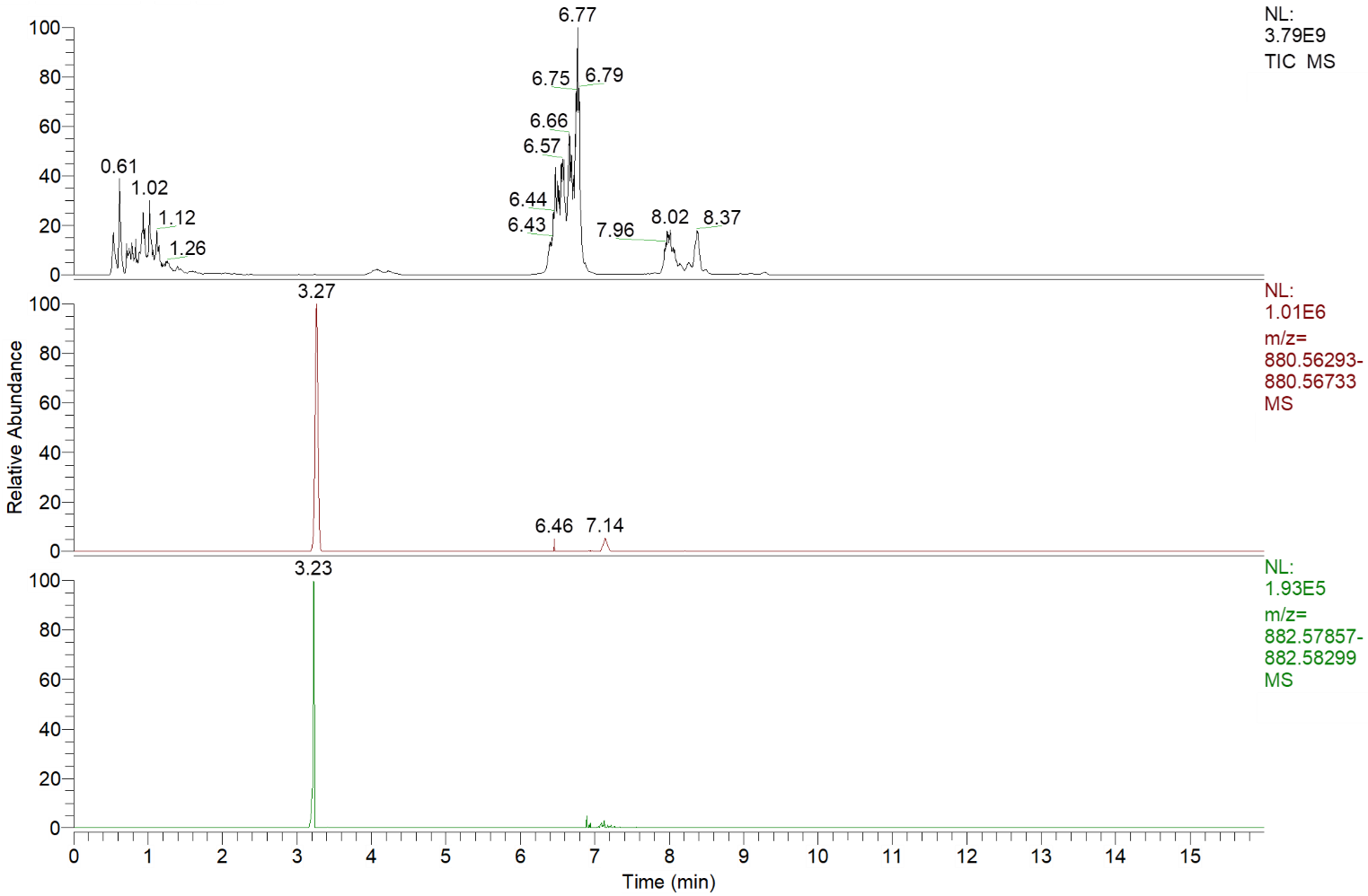


Fig. B.4. LC-HRMS TIC (top) measured for a plasma sample exposed to 250 µg/ml of HD. The EICs, extracted with 2.5 ppm accuracy, of HD-PC 34:2 (RT 3.27 min) and HD-PC 34:1 (3.23 min) are presented in the figures in the middle and at the bottom, respectively.


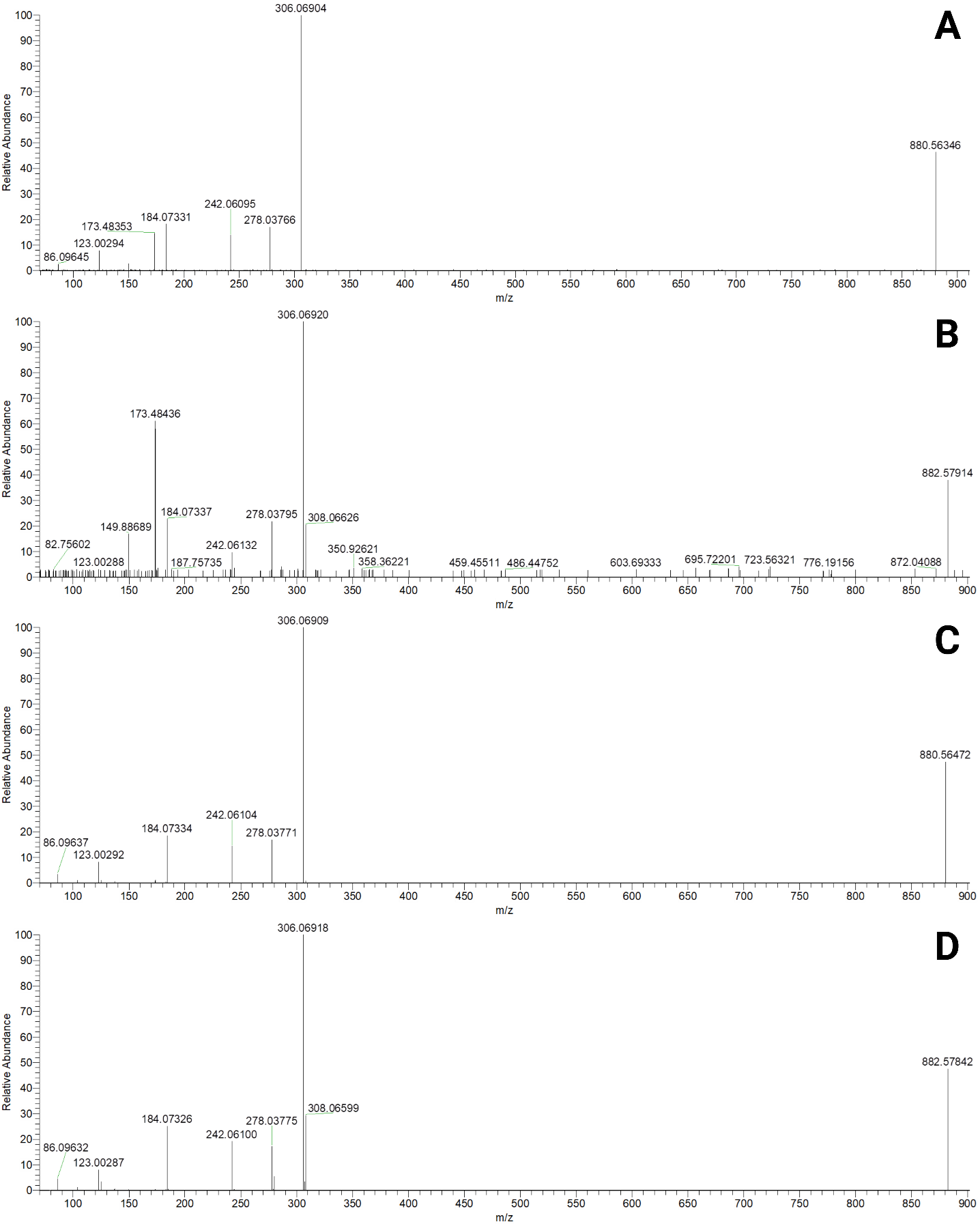


Fig. B.5. LC‒MS/HRMS spectra measured for HD-exposed plasma samples. Spectra A: HD-PC 34:2 in plasma exposed to 10 µg/ml HD, spectra B: HD-PC 34:1 in plasma exposed to 10 µg/ml HD, spectra C: HD-PC 34:2 in plasma exposed to 250 µg/ml HD, HD-PC 34:1 in plasma exposed to 250 µg/ml HD. Measured *m*/*z* for the parent ions and the fragments were within 5 ppm accuracy of the theoretical accurate *m*/*z*.
